# Toward Electrochromic Metallopolymers: Synthesis and Properties of Polyazomethines Based on Complexes of Transition-Metal Ions

**DOI:** 10.1021/acs.inorgchem.1c01249

**Published:** 2021-08-16

**Authors:** Sergiusz Napierała, Maciej Kubicki, Monika Wałęsa-Chorab

**Affiliations:** Faculty of Chemistry, Adam Mickiewicz University in Poznań, Uniwersytetu Poznańskiego 8, 61-614 Poznań, Poland

## Abstract

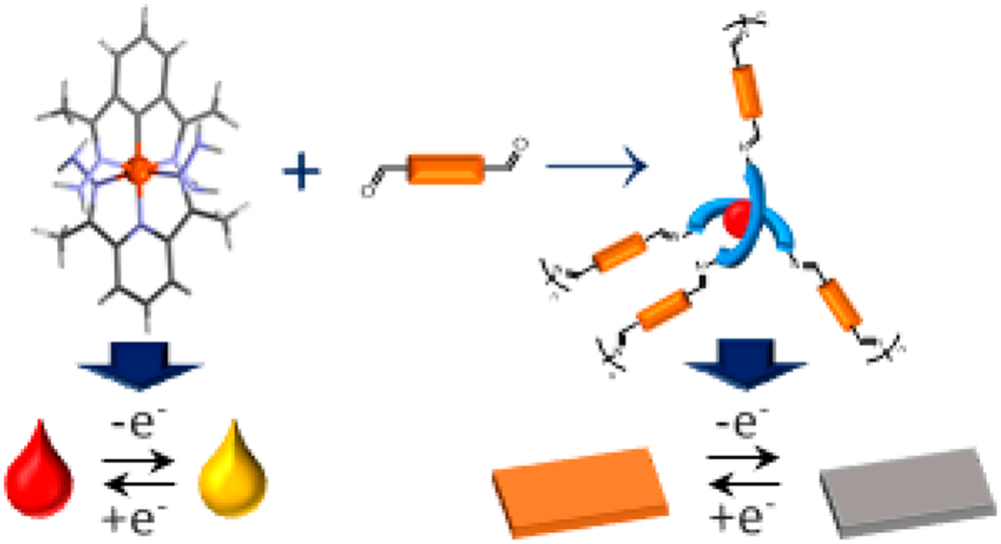

The tridentate ligand **L** and its complexes with transition-metal ions have been prepared and characterized. The polycondensation reactions of transition-metal complexes with different dialdehydes led to the formation of transition-metal-complex-based polyazomethines, which have been obtained by on-substrate polymerization, and their electrochemical and electrochromic performance have been investigated. The most interesting properties are exhibited by polymers of Fe(II) and Cu(II) ions obtained by the reaction of the appropriate complexes with a triphenylamine-based dialdehyde. Fe(II) polymer **P1** undergoes a reversible oxidation/reduction process and a color change from orange to gray due to the oxidation of Fe(II) to Fe(III) ions concomitant with the oxidation of the triphenylamine group. Its electrochromic properties such as long-term stability, switching times, and coloration efficiencies have been investigated, providing evidence of the utility of the on-substrate polycondensation reaction in the formation of thin films of electrochromic metallopolymers.

## Abstract 2

Polyazomethines containing transition-metal ions have been prepared by an on-substrate polymerization method and characterized in terms of their electrochemical and electrochromic properties. The polymer obtained by polycondensation of an iron(II)-based tetraamine and a triphenylamine-based dialdehyde was found to exhibit a color change from orange to gray and good electrochromic performance.

## Introduction

Polymeric complexes of transition-metal ions have been attracting interest in many scientific and technological fields in recent years due to their multiple applications.^1−3^ Complexes of transition-metal ions are known to be interesting materials for electrochromic applications, and the color change can be based on the redox reaction of the ligand molecule and/or the metallic center. The incorporation of transition-metal ions into the polymer backbone can also change the electrochromic properties of polymeric materials. This is the result of the formation of additional absorption bands that change the color of the material, such as metal to ligand (MLCT) or ligand to metal charge transfer (LMCT) bands as well as d–d transitions that are characteristic for transition-metal complexes.^4−6^

The synthesis of high-molecular-weight conjugated polymers containing transition metals has been often hampered by synthetic difficulties and/or a solubility problem. Metallosupramolecular polymers can be prepared by the complexation of metal ions with multidentate organic ligands or polymers^7−11^ as well as by cross-coupling reactions,^12^ radical polymerization,^13^ or electropolymerization.^14−18^ One of the methods of preparation of polymers is chemical linking of single or multiple kinds of monomers to form long chains, releasing water or a similar simple substance, called a polycondensation reaction. Hasanain et al. described a synthesis via a polycondensation reaction and the electrochromic characterization of a dinuclear ruthenium complex incorporated into the polyimide polymer main chains.^19^ The polymers exhibited interesting electrochromic properties, but they were not soluble in common organic solvents, which can hamper their processing. An example of the application of polycondensation to the preparation of metallopolymers was also demonstrated by the synthesis of electrochromic Co(III)- and Fe(III)-based metallosupramolecular polymers with aromatic azo ligands that conferred good solubility to the metal complex, and due to this the solubility issue has been overcome.^20^

Polyazomethines can also be prepared by an on-substrate method by heating monomers deposited onto the substrate under an acetic atmosphere.^21−23^ Polymers prepared in this way are obtained in the form of thin, insoluble layers on the substrate, and they cannot be characterized using conventional methods, such as gel permeation chromatography (GPC), but the insolubility of the polymer layer is desirable in case of application as an active layer in electrochromic devices. The miscibility of the organic layer with the electrolytic gel is problematic, because the electroactive layer can subsequently delaminate from the electrode, resulting in a limited device lifetime, poor device performance, localized color defects, and poor color contrast.^21^ An advantage of the on-substrate polymerization method is also the easy introduction of different functional groups, which in turn allows tuning of the polymer properties, and simple preparation and purification with only one byproduct, which is water.

Herein, we present the synthesis and characterization of polyazomethines containing complexes of transition-metal ions. Monomers for the synthesis of electrochromic polyazomethines by the on-substrate polymerization method are usually organic dialdehydes and diamines, but the use of transition-metal complexes has not been presented before. Complexes of transition-metal ions containing four amine groups that are able to undergo a condensation reaction with aldehydes have been obtained by the self-assembly of transition-metal ions with organic ligand 2,6-bis(1-hydrazonoethyl)pyridine (**L**). Complexes have been characterized by spectroscopic methods and X-ray analysis and have been used in polycondensation reactions with three different dialdehydes, leading to the preparation of several polyazomethines (**P1**–**P6**). An investigation of the electrochemical and electrochromic properties of the obtained materials allowed us to identify the key monomers that give polymers with the desired properties.

## Experimental Section

### General Considerations

Dialdehydes **4** and **5** have been prepared according to a previously reported procedure.^24^ All other reagents are commercially available and were used as received. Acetonitrile was dried by passing over neutral alumina followed by storage over 3 Å molecular sieves.^25^ NMR spectra were recorded on a Bruker UltraShield 300 MHz spectrometer and were calibrated against the residual protonated solvent signal (CDCl_3_, δ 7.24 ppm; *d*_6_-DMSO, δ 2.50 ppm). High-resolution ESI-MS spectra were run on a QTOF (Impact HD, Bruker) spectrometer in positive ion mode. XPS spectra were measured using a Specs UHV/XPS/SPM instrument. Al Kα was used as an X-ray source. The samples of complexes **1** and **2** were deposited on a piece of conducting carbon tape, while polyazomethines were measured as prepared on ITO-glass slides. The bonding energies were standardized using the C 1s peak at 285 eV, and the spectra were analyzed using Casa XPS software. IR spectra in the 4000–400 cm^–1^ region were measured in KBr pellets, obtained with a Perkin-Elmer 580 spectrophotometer, and are reported in cm^–1^. TGA analysis was carried out on a Netzsch TG 209 Libra instrument in the temperature range 30–600 °C under a nitrogen atmosphere at a heating rate of 10 °C min^–1^. SEM/EDX analysis was carried out with a Quanta 250 FEG, FEI high-resolution scanning electron microscope. AFM measurements were carried out on an Agilent 5500 instrument. Cyclic voltammetry was carried out in 0.1 M Bu_4_NPF_6_ acetonitrile solution using a three-electrode configuration (platinum working electrode in the case of measurements of electrochemical properties of complexes in solution or polymer-modified ITO-coated glass slides as the working electrode for measurements of electrochemical properties of polymers, Pt counter electrode, and Ag/Ag^+^ reference electrode) and a VSP Bio-Logic multichannel potentiostat. UV–vis–NIR absorption spectra were recorded on a Jasco V-770 spectrometer. Spectroelectrochemical measurements were carried out using a commercially available honeycomb electrode in the case of measurements of the properties of complexes in solution and polymer-modified ITO-coated glass slides, Pt counter electrode, and silver wire as a pseudoreference electrode for measurements of the electrochromic properties of polymers.

### X-ray Crystallography

Diffraction data were collected by the ω-scan technique, using graphite-monochromated Mo Kα radiation (λ = 0.71073 Å), at 100(1) K on a Rigaku Xcalibur four-circle diffractometer with an EOS CCD detector. The data were corrected for Lorentz–polarization as well as for absorption effects.^26^ Precise unit-cell parameters were determined by a least-squares fit of the reflections of the highest intensity, chosen from the whole experiment. The structures were solved with SHELXT^27^ and refined with the full-matrix least-squares procedure on *F*^2^ by SHELXL.^28^ All non-hydrogen atoms were refined anisotropically. Hydrogen atoms from NH_2_ groups were found in the difference Fourier maps and either kept in the positions found (**1** and **2**) or freely refined (**3**); all other hydrogen atoms were placed in idealized positions and refined as a “riding model” with isotropic displacement parameters set at 1.2 (1.5 for CH_3_) times the *U*_eq_ values of the appropriate carrier atoms. In the structure of **2** large voids have been found, filled with diffused electron density—probably a highly disordered solvent. As attempts to model the reasonable solvent model failed, the SQUEEZE procedure was successfully applied. Crystal data and data collection and structure refinement details of complexes **1**–**3** are summarized in Table S1.

### Synthesis

#### Ligand **L**

Ligand **L** has been obtained according to a previously reported method.^29^ 2,6-Diacetylpyridine (0.5 g, 3.07 mmol) was dissolved in absolute ethanol (10 mL). Afterward, excess hydrazine (5.0 mL) was added to the solution. The mixture was heated at 90 °C under an argon atmosphere overnight. The solution was concentrated and cooled with ice, and the obtained white crystals were filtered off, washed with a small amount of cold ethanol, and dried. Yield: 69% (0.40 g). ^1^H NMR (300 MHz, DMSO-*d*_6_): δ 7.72–7.66 (m, 2H), 7.58 (dd, *J* = 8.7, 6.7 Hz, 1H), 6.65 (s, 4H), 2.17 (s, 6H) ppm. ^13^C NMR (75 MHz, DMSO-*d*_6_): δ 155.3, 142.9, 135.8, 116.3, 9.5 ppm. HR-ESI-MS: (M + H)^+^ calcd 192.1244, found 192.1245; (M + Na)^+^ calcd 214.1064, found 214.1072. FT-IR (KBr): ν_as_(NH_2_) 3350; ν_s_(NH_2_) 3186; ν(C–H)_ar_ 3027; ν_as_(CH_3_) 2934; ν_s_(CH_3_) 2905; ν(C=N)_imin_ 1631; ν(C=C) 1599, 1566, 1522; ν(C=N) 1450, 1432, 1365; ν(C–N) 1287, 1249; ν(N–N) 1081; ρ(C–H) 1020, 991, 953, 806; γ(C–H) 737, 691, 643 cm^–1^. Anal. Calcd for C_9_H_13_N_5_ (191.23): C, 56.53; H, 6.85; N, 36.62. Found: C, 56.51; H, 6.88; N, 36.65.

#### Complex **1**

A mixture of ligand **L** (30 mg, 0.16 mmol) and Fe(BF_4_)_2_·6H_2_O (26.5 mg, 0.08 mmol) in a dichloromethane/acetonitrile mixture (6 mL, 1/1 v/v) was stirred at room temperature for 24 h. Then the solution was concentrated, and diethyl ether was added to precipitate the complex. The bloody red solid was centrifuged, washed with diethyl ether, and dried. HR-ESI-MS: [Fe**L**_2_(BF_4_)]^+^ calcd 525.1716, found 525.1710; [Fe**L**(**L**-H)]^+^ calcd 437.1608, found 437.1605; [Fe**L**_2_]^2+^ calcd 219.0840, found 219.0837. FT-IR (KBr): ν_as_(NH_2_) 3319; ν_s_(NH_2_) 3229; ν(C–H)_ar_ 3088; ν_as_(CH_3_) 2927; ν(C=N)_imin_ 1641; ν(C=C) 1600, 1562; ν(C=N) 1452, 1403, 1360; ν(C–N) 1284; ν(BF_4_^–^) 1049, 1032; ρ(C–H) 798; γ(C–H) 765, 746, 595 cm^–1^. Anal. Calcd for Fe(C_9_H_13_N_5_)_2_(BF_4_)_2_ (611.92): C, 35.33; H, 4.28; N, 22.89. Found: C, 35.35; H, 4.36; N, 22.85.

#### Complex **2**

A mixture of ligand **L** (30 mg, 0.16 mmol) and Cu(CF_3_SO_3_)_2_ (28 mg, 0.08 mmol) in a dichloromethane/acetonitrile mixture (6 mL, 1/1 v/v) was stirred at room temperature for 24 h. Then the solution was concentrated and diethyl ether was added to precipitate the complex. The green solid was centrifuged, washed with diethyl ether, and dried. HR-ESI-MS: [Cu**L**(CF_3_SO_3_)]^+^ calcd 402.9982, found 402.9984; [Cu(**L**-H)]^+^ calcd 253.0384, found 253.0384. FT-IR (KBr): ν_as_(NH_2_) 3321; ν_s_(NH_2_) 3218; ν_as_(CH_3_) 2953; ν(C=N)_imin_ 1647; ν(C=C) 1601, 1545; ν(C=N) 1473, 1460, 1382; ν(C–N) 1265; ν(CF_3_SO_3_^–^) 1242, 1225, 1027; ν(N–N) 1088; ν(BF_4_^–^) 1045, 1028; ρ(C–H) 805; γ(C–H) 758, 739, 634, 571, 516 cm^–1^. Anal. Calcd for Cu(C_9_H_13_N_5_)_2_(CF_3_SO_3_)_2_ (744.15): C, 32.28; H, 3.52; N, 18.82; S, 8.62. Found: C, 32.25; H, 3.59; N, 18.87; S, 8.59.

#### Complex **3**

A mixture of ligand **L** (30 mg, 0.16 mmol) and Cu(BF_4_)_2_ (19 mg, 0.08 mmol) in a dichloromethane/acetonitrile mixture (6 mL, 1/1 v/v) was stirred at room temperature for 24 h. Then the solution was concentrated and diethyl ether was added to precipitate the complex. The green solid was centrifuged, washed with diethyl ether, and dried. HR-ESI-MS: [Cu**L**(**L**-H)]^+^ calcd 444.1555, found 444.1564; [Cu(**L**-H)]^+^ calcd 253.0384, found 253.0388; [Cu**L**_2_]^2+^ calcd. 222.5814, found 222.5817. FT-IR (KBr): ν_as_(NH_2_) 3329; ν_s_(NH_2_) 3251; ν(C–H)_ar._ 3093; ν_as_(CH_3_) 2965; ν_s_(CH_3_) 2927; ν(C=N)_imin._ 1643; ν(C=C) 1603, 1549; ν(C=N) 1475, 1454, 1381; ν(C–N) 1287, 1259; ρ(C–H) 805; γ(C–H) 738, 676 cm^–1^. Anal. Calcd for C_9_H_13_N_5_ (191.23): C, 56.53; H, 6.85; N, 36.62. Found: C, 56.51; H, 6.88; N, 36.65. Anal. Calcd for Cu(C_9_H_13_N_5_)_2_(BF_4_)_2_ (619.62): C, 34.89; H, 4.23; N, 22.61. Found: C, 34.85; H, 4.28; N, 22.58.

#### Dialdehyde **6**

Dialdehyde **6** was prepared using a procedure modified from that described in the literature.^30^ A solution of 1,3-dibromobenzene (400 mg, 1.71 mmol), (4-formylphenyl)boronic acid (640 mg, 4.26 mmol), sodium carbonate (906 mg, 8.55 mmol), and tetrabutylammonium bromide (TBABr) (7 mg) in a toluene/water mixture (3/1 v/v, 10 mL) was degassed for 20 min under a flow of argon, and then tetrakis(triphenylphosphine)palladium(0) (200 mg, 0.17 mmol) was added under an argon atmosphere and the mixture was stirred and heated at 90 °C for 24 h. After the mixture was cooled to room temperature, dichloromethane (∼50 mL) was added and this mixture was extracted with water (3 × 30 mL) and brine. The organic layer was dried over MgSO_4_, the solvent was evaporated, and the crude product was purified by column chromatography on SiO_2_ using dichloromethane/hexane (4/5 v/v) as eluent. Dialdehyde **6** was obtained as a white solid (455 mg, 92%). ^1^H NMR (300 MHz, chloroform-*d*): δ 10.08 (s, 2H), 7.99 (d, *J* = 8.2 Hz, 4H), 7.87 (s, 1H), 7.81 (d, *J* = 8.2 Hz, 4H), 7.69 (dd, *J* = 6.8, 1.8 Hz, 2H), 7.60 (dd, *J* = 8.8, 6.4 Hz, 1H) ppm. ^13^C NMR (75 MHz, chloroform-*d*): δ 191.9, 146.8, 140.8, 135.6, 130.5, 129.9, 128.0, 127.6, 126.6 ppm. HR-ESI-MS: (M+H)^+^ calcd 287.1067, found 287.1079.

### Polymerization Procedure

#### On-Substrate Polymerization

To a solution of the complex of Fe(II) (**1**) or Cu(II) (**2**) (∼2.0 mg) solubilized in 0.5 mL of acetonitrile was added a solution of the appropriate dialdehyde (2.0 equiv) in 0.5 mL of dichloromethane. The mixtures with a total volume of 1 mL each were spray-coated for ∼3 min on cleaned ITO glass slides. The substrates were heated at 100 °C for 1 h under a saturated trifluoroacetic acid atmosphere. Afterward, the substrates were washed with a solution of triethylamine in dichloromethane and pure dichloromethane to remove any unreacted monomers and dried on air.

#### Polymerization in Solution

To a solution of complex **1** or **2** (∼20 mg) in acetonitrile were added a solution of the appropriate dialdehyde (2 equiv) in chloroform and a catalytic amount of TFA (∼5 mol %), and the mixture was stirred and heated at 70 °C for 24 h. Afterward triethylamine was added to neutralize the acid and the precipitated polymers were filtered off, washed with acetonitrile and chloroform to remove unreacted monomers, and dried on air.

##### Polymer **P1**

Yield: 67%. FT-IR (KBr): ν(NH_2_) 3320, 3226, 3200; ν(C–H)_ar_ 3032; ν_as_(CH_3_) 2963; ν_s_(CH_3_) 2922; ν(C=O) 1687; ν(C=N)_imin_ 1628, 1621; ν(C=C) 1589, 1549, 1521; ν(C=N) 1490, 1422, 1399, 1378; ν(C–N) 1324, 1276, 1263; ν(N–N) 1185; ν(BF_4_^–^) 1049, 1026; ρ(C–H) 1083, 871, 815, 799; γ(C–H) 740, 718, 695 cm^–1^.

##### Polymer **P2**

Yield: 72%. FT-IR (KBr): ν(NH_2_) 3262; ν(C–H)_ar_ 3034; ν_as_(CH_3_) 2965; ν_s_(CH_3_) 2922; ν(C=O) 1700; ν(C=N)_imin_ 1634, 1626; ν(C=C) 1591, 1544, 1523; ν(C=N) 1491, 1455, 1419, 1404, 1364; ν(C–N) 1324, 1275, 1186, 1179, 1156, 1144, 1105; ν(CF_3_SO_3_^–^) 1261, 1223, 1029, 1004; ν(N–N) 1150; ρ(C–H) 850, 814, 754; γ(C–H) 740, 696, 635, 515 cm^–1^.

##### Polymer **P3**

Yield: 63%. FT-IR (KBr): ν(NH_2_) 3373; ν_as_(CH_3_) 2975; ν_s_(CH_3_) 2900; ν(C=O) 1701; ν(C=N)_imin_ 1637, 1617; ν(C=C) 1591, 1537; ν(C=N) 1498, 1486, 1443, 1413, 1399, 1378; ν(C–N) 1310, 1276, 1198; ν(N–N) 1179; ν(BF_4_^–^) 1052, 1032; ρ(C–H) 1142, 971, 870, 834, 797; γ(C–H) 742, 718, 703, 519 cm^–1^.

##### Polymer **P4**

Yield: 51%. FT-IR (KBr): ν(C–H)_ar_ 3065; ν_as_(CH_3_) 2959; ν_s_(CH_3_) 2922; ν(C=O) 1701; ν(C=N)_imin_ 1652, 1639; ν(C=C) 1600, 1567, 1539; ν(C=N) 1496, 1450, 1412, 1362; ν(C–N) 1310, 1275, 1177, 1112; ν(CF_3_SO_3_^–^) 1260, 1223, 1029; ν(N–N) 1154; ρ(C–H) 971, 836, 801; γ(C–H) 742, 728, 697, 637 cm^–1^.

##### Polymer **P5**

Yield: 69%. FT-IR (KBr): ν(NH_2_) 3277, 3166; ν(C–H)_ar_ 3044; ν_as_(CH_3_) 2991; ν_s_(CH_3_) 2922; ν(C=O) 1700; ν(C=N)_imin_ 1636; ν(C=C) 1594, 1553; ν(C=N) 1476, 1433, 1397, 1383; ν(C–N) 1308, 1282, 1181, 1142; ν(N–N) 1084; ν(BF_4_^–^) 1050, 1032; ρ(C–H) 875, 835, 793; γ(C–H) 702, 698, 608, 518 cm^–1^.

##### Polymer **P6**

Yield: 61%. FT-IR (KBr): ν(C–H)_ar_ 3060, 3029; ν_as_(CH_3_) 2970; ν_s_(CH_3_) 2922; ν(C=O) 1697; ν(C=N)_imin_ 1660, 1631; ν(C=C) 1604, 1546, 1516; ν(C=N) 1477, 1457, 1410, 1366; ν(C–N) 1308, 1274, 1179, 1114; ν(CF_3_SO_3_^–^) 1261, 1223, 1029, 1007; ν(N–N) 1155; ρ(C–H) 960, 976, 837, 839, 793; γ(C–H) 738, 700, 637, 610, 572, 555, 517 cm^–1^.

## Results and Discussion

**Figure 1:**
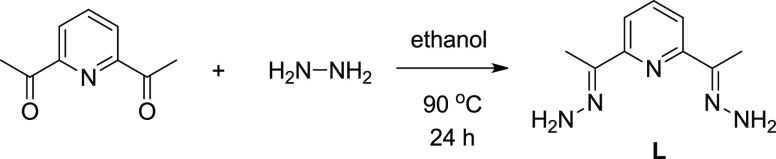
Synthesis of ligand **L**.

Ligand **L** has been prepared in a condensation reaction between 2,6-diacetylpyridine and hydrazine monohydrate,^29^ as outlined in Figure 1.

The backbone of ligand **L** contains a structural unit similar to that of terpyridine, and it has been designed to act as a tridentate donor ligand in reactions with transition-metal ions to form complexes of 1:2 metal to ligand stoichiometry that contain four uncoordinated amine groups able to undergo a condensation reaction with other functional groups. The ligand is known to be potentially pentadentate,^31^ but it mainly acts as a tridentate ligand.^29,32−34^ The reactions of ligand **L** with iron(II) and copper(II) salts were carried out in a dichloromethane/acetonitrile mixture (1/1 v/v) at room temperature for 24 h. Complexes have been obtained as colored solids by precipitation using diethyl ether, isolated and characterized. The 1:2 metal:ligand stoichiometry of obtained complexes has been confirmed by high-resolution electrospray ionization mass spectra. For example, the signals at *m*/*z* 219.0837 and 525.1710 in the ESI-MS spectra of complex **1** have been assigned to [Fe**L**_2_]^2+^ and [Fe**L**_2_](BF_4_)^+^, respectively, and clearly indicate the formation of a complex with a 1:2 stoichiometry. In the ESI-MS spectra signals at *m*/*z* 437.1605 and 444.1564 are also present, which have been assigned to molecular cations with a 1:2 metal:ligand stoichiometry in which one of the ligand molecules is deprotonated, forming the molecular cations [Fe**L**(**L**-H)]^+^ and [Cu**L**(**L**-H)]^+^, respectively. The infrared spectra of the complexes of transition-metal ions provide useful information about the coordination of the functional groups upon the formation of the complexes in the solid state. The IR spectra of the products have been assigned by comparison with the stretching frequencies of the free ligand **L**. The peaks in the IR spectra of free ligand **L** were consistent with those observed for similar hydrazine derivatives.^35,36^ The ligand **L** and its complexes with transition-metal ions show characteristic IR bands in the range 1647–1630 cm^–1^, due to the stretching vibration of the imine C=N bond (Figures S1–S4).^37^ The weak bands in the spectra of the ligand **L** at around 3080 and 3026 cm^–1^ were attributed to an aromatic C–H bond stretching vibration, whereas the bands at 2933 and 2907 cm^–1^ were assigned to the C–H stretching vibration of the methyl group. The two bands in the range of 3150–3350 cm^–1^ in the FT-IR spectra of both the ligand and the complexes were assigned to asymmetric and symmetric stretches of the amine group, confirming that −NH_2_ groups in the transition-metal complexes still exist.

**Figure 2:**
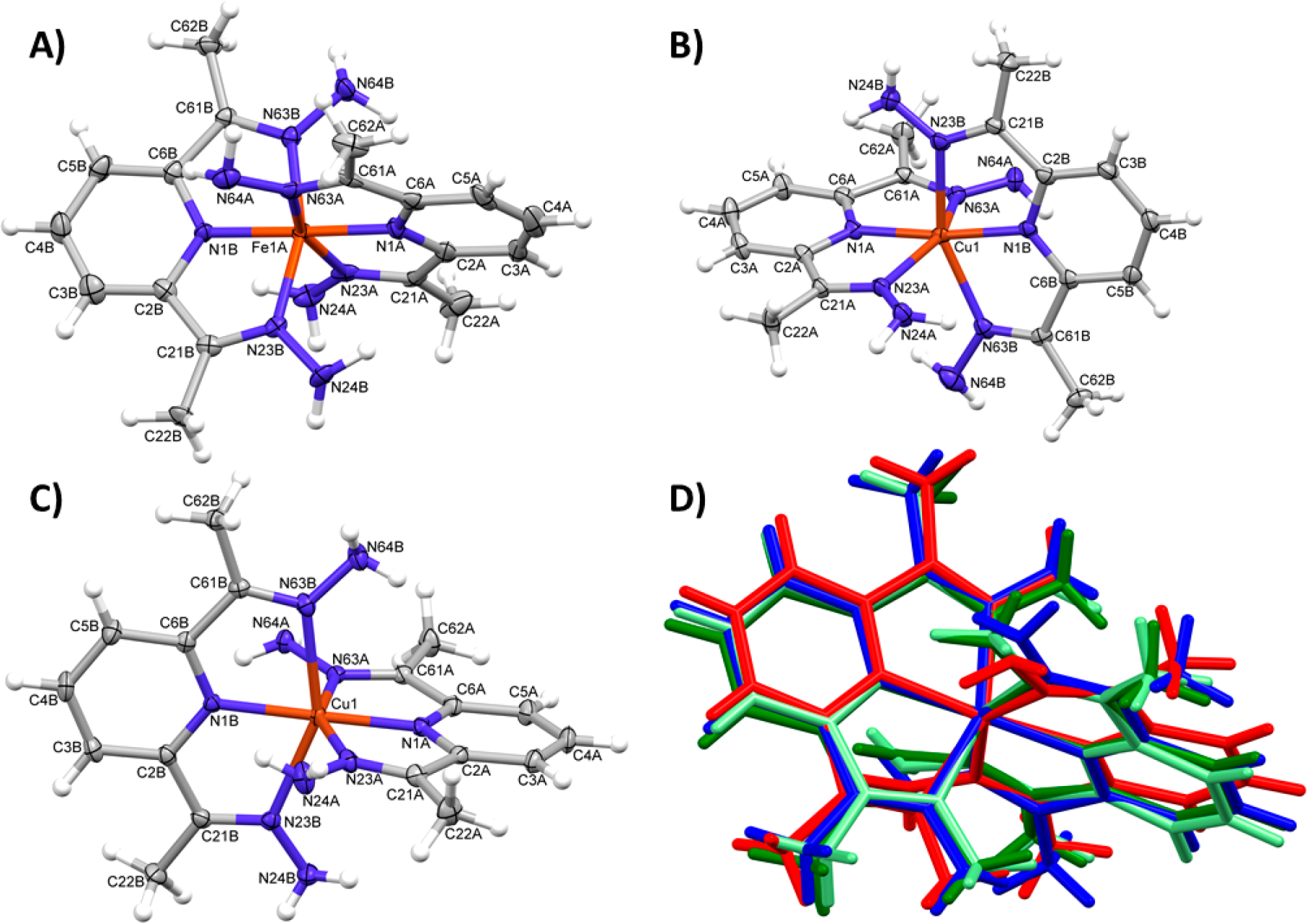
Perspective views of one of the symmetry-independent cationic complexes of **1** (A) and cations observed in the structures of **2** (B) and **3** (C). Ellipsoids are drawn at the 50% probability level, and hydrogen atoms are shown as spheres of arbitrary radii. (D) Comparison of four dications, where the metal and coordinated N atoms were fitted onto one another. Color codes: **1A**, green; **1B**, pale green; **2**, red; **3**, blue (the asymmetric part of the unit cell of **1** contains two symmetry-independent molecules).

The exact crystal structures of complexes **1**–**3** have been confirmed by X-ray crystallography. Single crystals appropriate for an X-ray analysis have been obtained by slow diffusion of diethyl ether into acetonitrile solutions of complexes. Figure 2A–C shows the perspective views of the dications **1**–**3**, respectively; Table 1 gives the relevant geometrical parameters. All complexes have an M**L**_2_ structure, and the overall shapes and coordinations of all four dications (in the structure of **1** there are two symmetry-independent complexes) are very similar (Figure 2D). The coordination of the metal cation is best described as distorted octahedral (cf. Table 1), with a quite linear N(pyridine)–M–N(pyridine) angle.

**Table 1:** Relevant Geometrical Parameters (Å, deg) with su Values in Parentheses

	**1A** (M = Fe)	**1B** (M = Fe)	**2** (M = Cu)	**3** (M = Cu)
M1–N1	1.874(5)	1.878(5)	1.960(2)	1.9360(16)
	1.882(5)	1.884(5)	1.965(2)	1.9749(16)
				
M1–N23	1.955(5)	1.963(5)	2.186(2)	2.1344(17)
	1.955(6)	1.967(5)	2.188(2)	2.1396(17)
				
M1–N63	1.959(5)	1.980(5)	2.206(2)	2.2710(17)
	1.978(5)	1.988(5)	2.214(2)	2.2802(17)
				
angles	176.6(2)	176.9(2)	173.31(9)	178.80(7)
	160.0(2)	159.1(2)	153.31(8)	155.43(6)
	159.4(2)	159.0(2)	151.24(8)	151.77(6)

In the crystal structures the cations, anions, and solvent molecules make three-dimensional networks by means of Coulombic interactions, hydrogen bonds between N–H groups and counterion F or O atoms, and van der Waals contacts.

**Figure 3:**
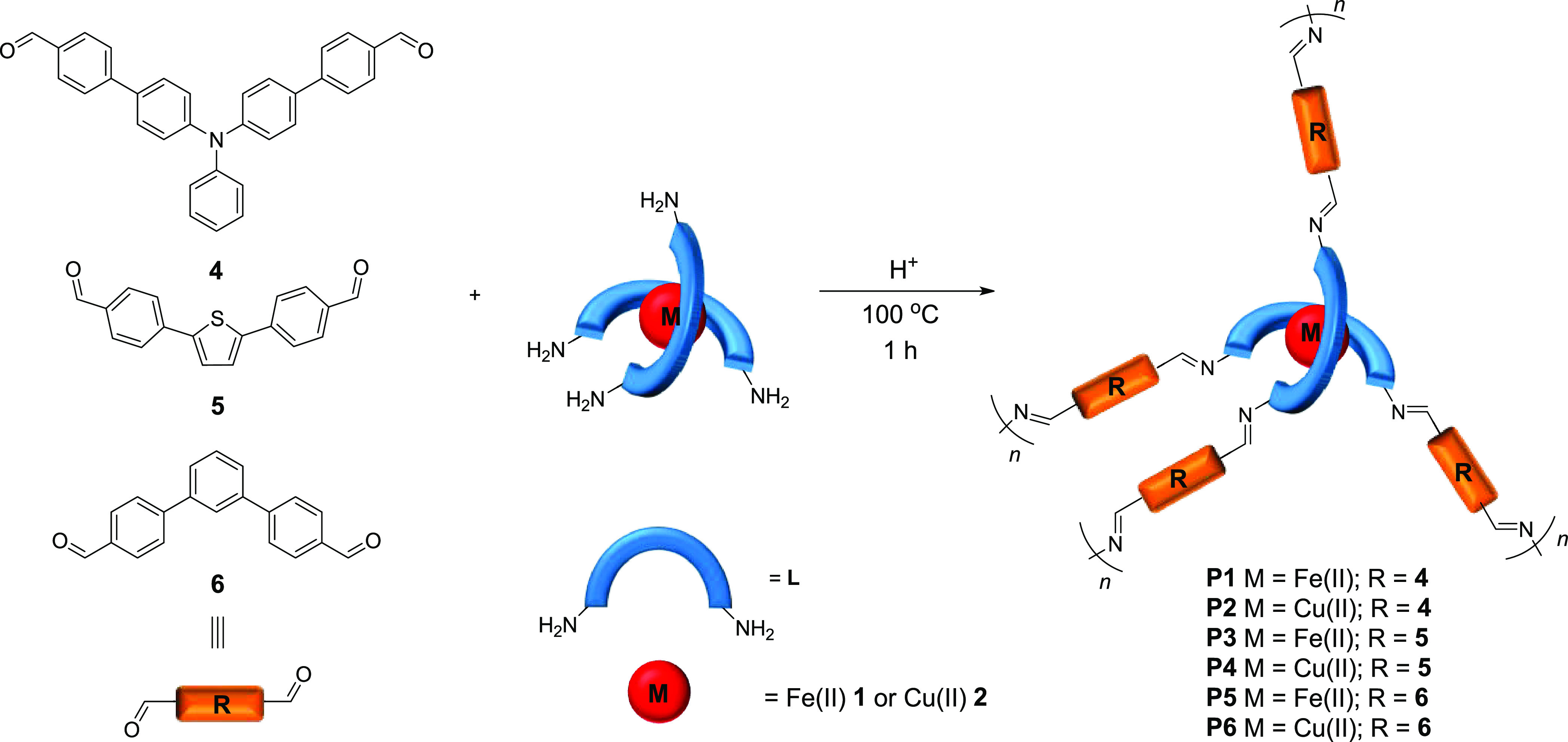
Scheme showing the preparation of transition-metal-complex-based polyazomethines **P1**–**P6**.

The polycondensation reactions between various dialdehydes **4**–**6** and complexes **1** and **2** being tetraamine monomers shown in Figure 3 led to the preparation of polyazomethines containing transition-metal ions.

The dialdehydes have been selected to investigate the influence of the central groups on the electrochemical and electrochromic properties of the obtained polymers. The dialdehyde **6** does not contain an electrochromic group, while dialdehydes **4** and **5** contain electroactive triphenylamine and thiophene groups, respectively, which are known to affect the electrochromic properties of polymers.^24^

To evaluate the thermal stability of ligand **L** and its complexes and to confirm the stability of the compounds under on-substrate polymerization conditions, a thermogravimetric analysis (TGA) was carried out. The TGA measurements were done in a temperature range of 30–600 °C under a nitrogen atmosphere at a heating rate of 10 °C min^–1^. It was found that ligand **L** was stable up to 180 °C before it underwent any significant decomposition (Figure S5), whereas complexes with transition-metal ions were found to decompose above 215 °C (**1**), 190 °C (**2**), and 310 °C (**3**) (Figures S6–S8). The small weight loss in the temperature range of 30–100 °C observed in the case of complexes of transition-metal ions has been assigned to the loss of solvent molecules.

The polycondensation of complexes **1** and **2** with dialdehydes was done by an on-substrate polymerization method.^21,22^ To do this, a mixture of the monomers in a 1:2 transition-metal complex:dialdehyde molar ratio was manually spray coated onto ITO-coated glass slides and the plates were heated at 100 °C for 1 h under a saturated trifluoroacetic acid atmosphere. Afterward, the substrates were rinsed with dichloromethane solution containing triphenylamine and pure dichloromethane to neutralize the acid and remove any unreacted monomers and low-molecular-weight oligomers. To characterize the polymers by TGA analysis and infrared spectroscopy, polymers were also obtained in solution. To do this, to a solution of complex **1** or **2** in acetonitrile was added a solution of the appropriate dialdehyde (2 equiv) in chloroform. Then a catalytic amount of TFA (∼5 mol %) was added to the mixture of monomers and the solution was stirred and heated at 70 °C for 24 h. Afterward triethylamine was added to neutralize the acid and precipitated polymers were filtered off, washed with acetonitrile and chloroform to remove unreacted monomers, and dried on air. Polymers **P1**–**P6** were found to exhibit thermal stability similar to that of complexes of transition-metal ions that were used as monomers (Figures S9–S14). Decomposition of the iron-based polymers **P1**, **P3**, and **P5** was found to occur above 300 °C, whereas copper-based polymers **P2**, **P4**, and **P6** decompose at temperatures above 200 °C. The small weight losses at temperatures below 200 °C have been assigned to the loss of solvent molecules adsorbed on the surface of polymer and/or occluded in the polymers. In the FT-IR spectra, due to the existence of two imine bonds in different surroundings in the polymers, two characteristic bands can be attributed to the imino groups (Figures S15–S20). In the spectra there are also very weak signals in the ranges of 3350–3200 and 1690–1670 cm^–1^ assigned to amine NH_2_ and carbonyl C=O groups, respectively, that indicate the existence of unreacted end groups of the polymers.

The composition of polymers obtained by on-substrate polymerization was analyzed using X-ray photoelectron spectroscopy (XPS). As seen in Figure S21, XPS survey spectra of complex **1** and its polymers contain peaks of the core levels C 1s, N 1s, Fe 2p, B 1s, F 2p, and S 2p in case of polymer **P3**, whereas in case of complex **2** and its polymers core levels of C 1s, N 1s, Cu 2p, S 2p, O 2p, and F 2p have been detected (Figure S22). Additionally, in the case of polymers **P1**–**P6** the XPS survey spectra contain In, Sn, Si, and O core levels of the ITO support. The 2p Fe and 2p Cu peaks for polymers **P1** and **P2** appeared at 703.96 and 932.94 eV for Fe 2p_3/2_ and Cu 2p_3/2_, respectively, which was lower than those of complexes **1** and **2** (708.47 and 935.04 eV for Fe 2p_3/2_ and Cu 2p_3/2_, respectively) (Figure S23). These results suggest that metal ions become more cationic in polyazomethines,^38^ most likely due to the lower electronegativity of imine nitrogen atoms in comparison to amine nitrogen atoms that increases the positive charge on metal ions.

**Figure 4:**
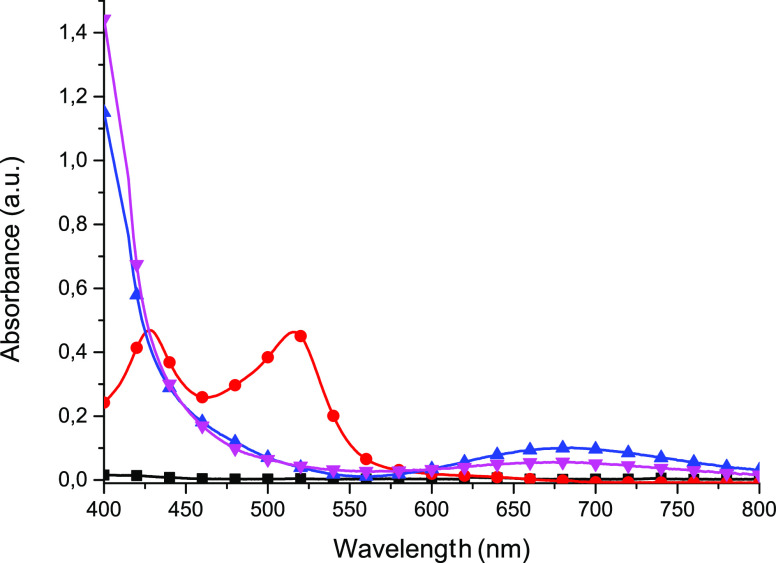
Absorption spectra of ligand **L** (■ black) measured in dichloromethane and complexes **1** (● red), **2** (▲ blue) and **3** (▼ magenta) in acetonitrile.

The obtained compounds have been characterized in terms of their electrochemical and electrochromic properties. First, absorption spectra in the visible region of ligand **L** and its complexes in a dichloromethane solution in the case of ligand **L** and in acetonitrile solutions for the complexes have been recorded (Figure 4).

Ligand **L** exhibits no absorption in the visible range with a strong absorption below 340 nm (Figure S24). The Fe(II) complex **1** exhibits two absorption bands in the visible region with maxima at 516 and 428 nm, which can be assigned to metal to ligand charge transfers (MLCTs). Such an electronic behavior has already been observed for homoleptic iron-based compounds with pyridine-based ligands, and it can be explained by the first MLCT band at 428 nm involving the ligand subunit whose energy remained unchanged whatever the substitution and the second MLCT band at lower energy involving the central pyridine ring, which is sensitive to electronic tuning on this ring.^39,40^ In the case of Cu(II) complexes weak d–d bands at 685 and 677 nm for complexes **2** and **3**, respectively, are responsible for the green color of the copper(II) complexes.

To examine the influence of the electrochromic properties of transition-metal complexes on polyazomethines, first the electrochemical and spectroelectrochemical measurements of complexes were carried out in solution. The electrochemical properties of the compounds were examined using cyclic voltammetry. The measurements were made in an anhydrous and deaerated 0.1 M solution of tetrabutylammonium hexafluorophosphate in acetonitrile as the supporting electrolyte.

Complex **1**, which contains an Fe(II) ion was found to undergo the reversible oxidation/reduction process Fe(II) ↔ Fe(III) with an anodic peak potential (*E*_pa_) of +1.27 V and a cathodic peak potential (*E*_pc_) of +1.17 V. In the case of complex **2**, containing a Cu(II) ion, an irreversible reduction peak has been observed at the cathodic peak potential *E*_pc_ = −0.41 V associated with the reduction process Cu(II) → Cu(I). In the anodic scan a peak for the irreversible oxidation of Cu(I) ions into Cu(II) ions has been observed at an anodic peak potential of +0.33 V. **2** has been chosen as an example of a Cu(II)-based complex for the examination of electrochemical and electrochromic properties due to the more intense green color in comparison with **3** in solution at the same concentration of the complex.

**Figure 5:**
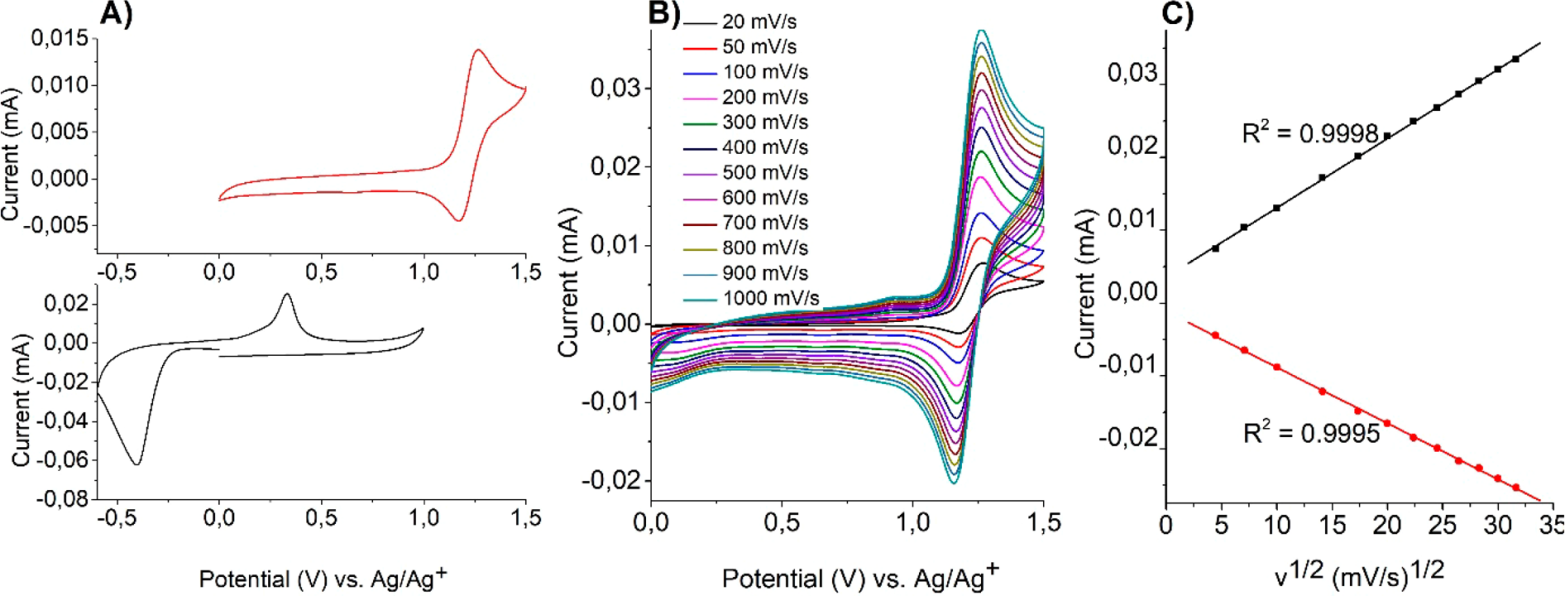
(A) Cyclic voltammetry of complexes **1** (red) and **2** (black) measured in anhydrous and deaerated 0.1 M solutions of TBAPF_6_ in acetonitrile at a scan speed of 100 mV/s. (B) CV profiles of complex **1** at different scan rates. (C) Linear dependence of the peak currents on the square root of the scan rate for complex **1**.

Due to the irreversible character of oxidation/reduction of the metallic center in complexes of Cu(II) ions, the characterization of the redox process was carried out for an Fe(II) complex. It was done by recording cyclic voltammograms at different scan rates and examining the dependence of the peak current on the scan rate (Figure 5B). In case of complex **1** in solution it was found that both anodic and cathodic peak currents are linearly dependent on the square root of the scan rate with excellent linear correlation coefficients (*R*^2^) of 0.9998 and 0.9995 for *i*_pa_ and *i*_pc_, respectively (Figure 5C). This indicates a reversible electron transfer process involving freely diffusing redox species.

**Figure 6:**
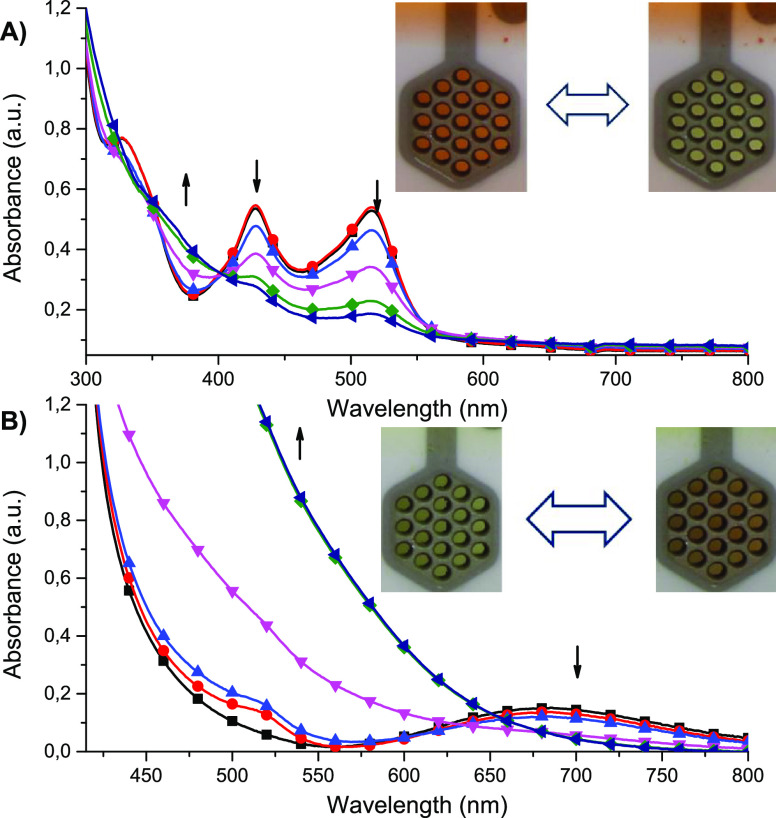
Spectroelectrochemistry of (A) complex **1** measured in an anhydrous and deaerated 0.1 M solution of TBAPF_6_ in acetonitrile as the supporting electrolyte with applied potentials of 0 (● red), 0.9 (■ black), 1.0 (▲ blue), 1.1 (▼ magenta), 1.2 (⧫ green), and 1.3 V (◀ navy) versus Ag/Ag^+^ and (B) complex **2** measured in an anhydrous and deaerated 0.1 M solution of TBAPF_6_ in acetonitrile as the supporting electrolyte with applied potentials of 0 (■ black), −0.1 (● red), −0.2 (▲ blue), −0.3 (▼ magenta), −0.4 (⧫ green), and −0.5 V (◀ navy) versus Ag/Ag^+^.

The oxidation/reduction of metallic centers was accompanied by an intrinsic color change that was tracked by spectroelectrochemistry. It combines both electrochemistry and spectroscopy and allows us to track the color changes *in situ*. Complex **1** was found to change from red to yellow as a result of the electrooxidation of Fe(II) ions into Fe(III) ions (Figure 6A). This was accompanied by a gradual decrease of the MLCT bands at 516 and 428 nm and increase of the absorbance in the range of 350–400 nm that can be attributed to LMCT ofthe Fe(III) complex.^41,42^ The sharp isosbestic point at 402 nm confirms that only oxidized and reduced species are present in the solution and no side products of electrooxidation are formed. In case of Cu(II) complex **2** the observed color change was from green to red-brown as a result of the electroreduction Cu(II) → Cu(I). The color change was connected with the disappearance of the d–d band at 685 nm accompanied by an increase in the absorbance in the range below 600 nm (Figure 6B).

**Figure 7:**
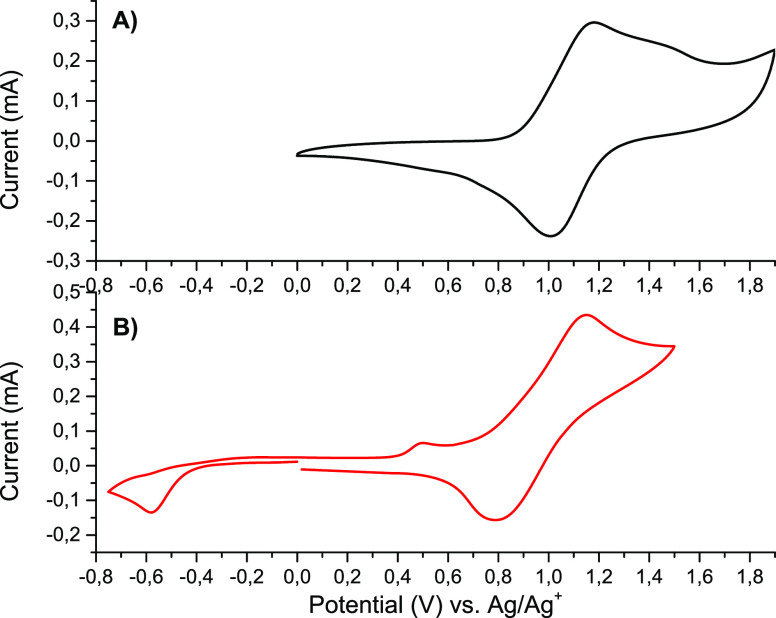
Cyclic voltammograms of (A) polymer **P1** and (B) polymer **P2** measured in an anhydrous and deaerated 0.1 M solution of TBAPF_6_ in acetonitrile at a scan rate of 20 mV/s.

The electrochemical and spectroelectrochemical properties of polyazomethines were investigated using polymer-modified ITO-coated glass electrodes. Polymers **P1** and **P2** obtained by polycondensation of complexes of Fe(II) and Cu(II), respectively, with a triphenylamine-based dialdehyde were found to undergo oxidation/reduction processes of both metallic centers and the triphenylamine group (Figure 7).

The oxidation potentials of polymer **P1** were found to be highly dependent on the scan rate. At a scan rate of 20 mV/s in the anodic part of the cyclic voltammogram an oxidation peak in the range of +1.0 to +1.6 V has been observed (Figure 7A) that can be fitted with two fitting functions, giving peaks with maxima at +1.14 and +1.42 V (Figure S25). The first oxidation potential was associated with the oxidation of the triphenylamine group, while the second peak originates from the oxidation of Fe(II) ions. With an increase in the scan speed the shift of the first oxidation potential to more positive values has been observed and it overlaps with the second oxidation peak, and at a scan speed of 1000 mV/s it appears at a potential of +1.7 V (Figure S26). Similarly the reverse peak in the cathodic part of the cyclic voltammograms shifts toward more negative potential upon an increase in the scan rate from +1.0 V at a scan rate of 20 mV/s to +0.5 V at a scan rate of 1000 mV/s. Indeed, the peak to peak CV separation (Δ*E*p) increases with the scan rate, which indicates the presence of electrochemical irreversibility and a quasi-reversible redox process;^43^ this is because the peak potentials of the forward and backward scans are always shifted by a non-negligible voltage value, while a true reversible process should feature the same (reduction and oxidation) potential values.^44^ The linear behavior (*R*^2^ = 0.9944) of the peak current as a function of the scan rates (Figure S27) indicates that the redox events occur at the electrode surface, demonstrating that the polymer was successfully attached to the ITO electrode.^43^ In the cyclic voltammogram of polymer **P2** an irreversible oxidation wave at *E*_pa_ = +0.49 V connected with the electrooxidation of Cu(I) to Cu(II) ions and a quasi-reversible redox event with a half-wave potential of *E*_1/2_ = +0.97 V (*E*_pa_ = +1.15 V and *E*_pc_ = +0.79 V) associated with the redox reaction of triphenylamine group have been observed (Figure 7B). Additionally in the cathodic part of the voltammogram irreversible reduction of Cu(II) to Cu(I) ions at a potential of −0.58 V has been detected. The oxidation and reduction potentials of polymer **P2** are shifted to more positive and more negative potentials, respectively, in comparison to complex **2**, indicating that the metal centers in polymers are becoming hard to oxidize or reduce. This is consistent with an increase in the resistance of the film.

Polymers **P3** and **P4** exhibited redox peaks from electrochemical reactions of both the metallic centers and the thiophene group (Figure S28). An irreversible oxidation process of Fe(II) ions in polymer **P3** was observed at *E*_pa_ = +1.61 V, whereas irreversible oxidation and reduction processes based on the redox reaction of Cu(II) ions in the case of polymer **P4** were observed at +0.85 and −0.62 V, respectively. Additionally, irreversible oxidation waves at *E*_pa_ = +1.99 and +2.12 V for polymers **P3** and **P4**, respectively, were observed that are consistent with oxidation of the thiophene moiety.^45,46^ In the case of polymers **P5** and **P6** obtained by the polycondensation of transition-metal ions complexes with dialdehyde **6**, which does not contain any electroactive group, we expected to obtain thin films that exhibit oxidation/reduction processes based on the redox reaction of transition-metal ions, but no oxidation/reduction peaks were observed in the investigated potential window. This was probably due to the high insulating character of the obtained polyazomethines.

**Figure 8:**
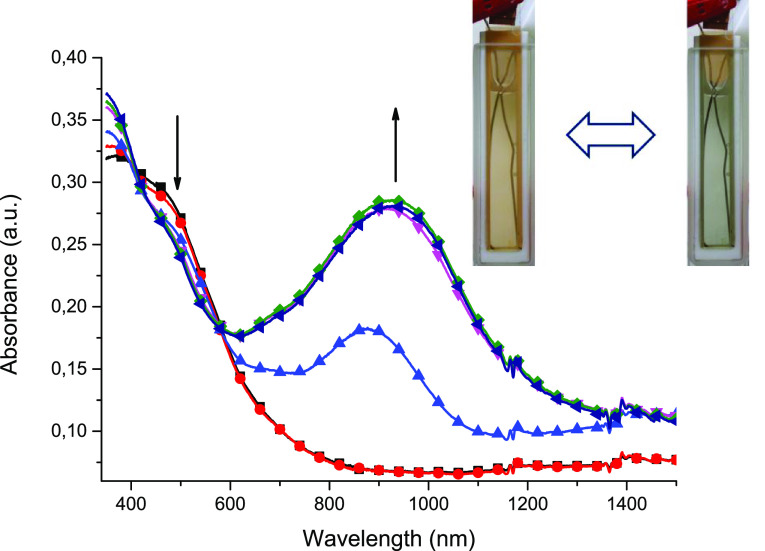
Spectral changes of polymer **P1** measured in anhydrous and deaerated 0.1 M TBAPF_6_ in acetonitrile as the supporting electrolyte by applying 0 (■ black), +1.2 (▲ blue), +1.3 (▼ magenta), +1.4 (⧫ green), +1.5 (◀ navy), and −0.1 V (● red) potentials versus an Ag/Ag^+^ reference electrode held for 30 s per potential. Insert: photographs of the original (left) and electrooxidized (right) **P1**.

Due to the best electrochemical properties of polymers **P1** and **P2** containing a triphenylamine group, they were chosen for further investigation of their electrochromic properties. The polymer **P1** was found to undergo a color change from orange to gray as a result of electrooxidation. This was accompanied by a decrease in the MLCT band at 475 nm and the formation of a band below 400 nm, which was assigned to a ligand to metal charge transfer (LMCT) band of the Fe(III) complex,^47,48^ as well as the formation of new, broad band partially in the NIR region with a maximum at 930 nm (Figure 8), which was connected with the formation of a radical cation on the triphenylamine group in azomethines.^49,50^

The isosbestic point at 580 nm confirms that only two forms, oxidized and neutral forms, are present, which indicates that oxidation of Fe(II) to Fe(III) is concomitant with the oxidation of the triphenylamine group. The color change was reversible, and after applicataion of a slightly negative potential (−0.1 V), neutralization of the triphenylamine group and reduction of Fe(III) to Fe(II) occur, which resulted in the restoration of the initial UV–vis–NIR spectra.

**Figure 9:**
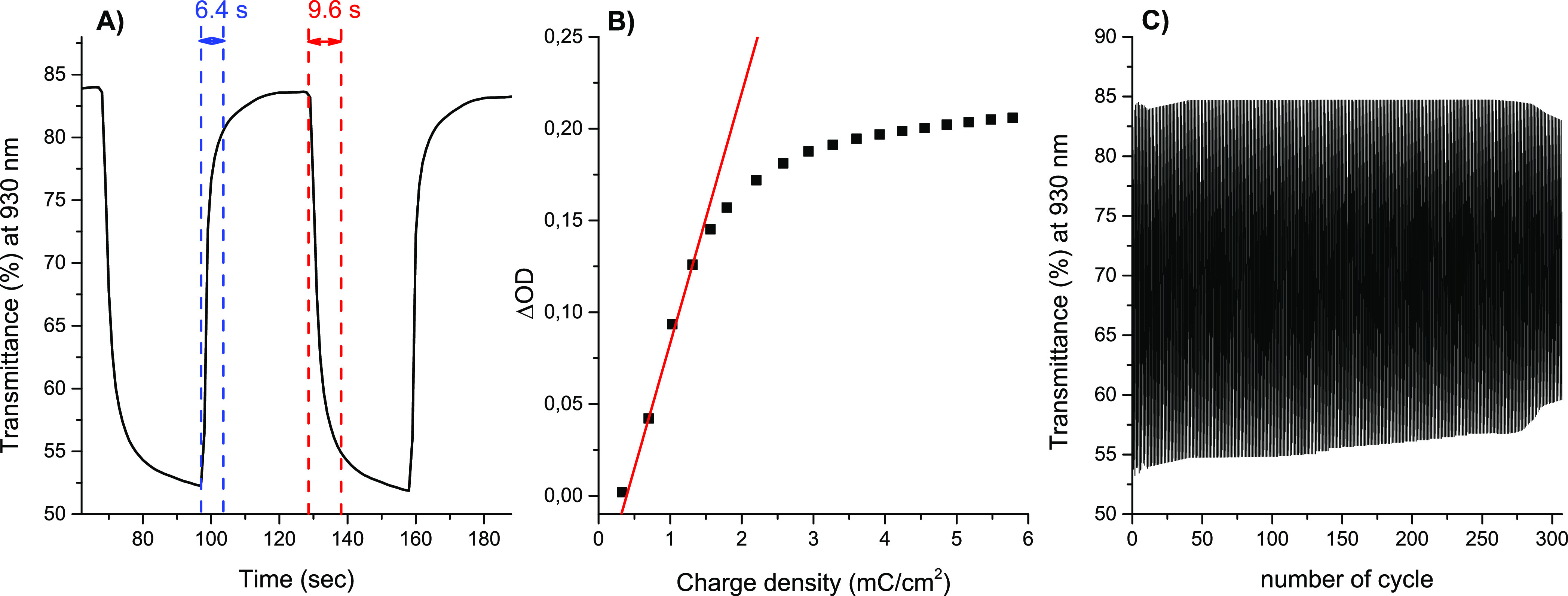
(A) Coloration and bleaching times of polymer **P1**. (B) Plot of the optical density of **P1** at 930 nm versus charge density. The CE value was calculated from the slope of the line fitted to the linear region of the curve (red line). (C) Electrochromic stability of the polymer **P1** immobilized onto an ITO glass slide electrode measured in an anhydrous and deaerated 0.1 M solution of TBAPF_6_ in acetonitrile by switching between +1.5 and −0.1 V in 10 s intervals, monitored at 930 nm.

To investigate the time that is required for the polymer to change in color from orange to gray, switching between +1.5 and −0.1 V was carried out 10 times in 30 s intervals to ensure the completion of the electrochromic reaction and the changes in transmittance were monitored at the absorption maximum of the oxidized state at 930 nm (Figure S29). The switching times, coloration (*T*_c,90_) and bleaching (T_b,90_) times, were calculated as the times required to reach 90% of the final change in transmittance between neutral and oxidized states (Figure 9A), and they were found to be 9.6 and 6.4 s for the coloring and bleaching steps, respectively.

The maximum transmittance difference between the oxidized and reduced states was calculated to be 31%, and it did not change after 10 switching cycles. Coloration efficiency is an important characteristic for electrochromic materials, and it is a proportionality factor between the optical absorbance change of an electrochrome at a designated wavelength (optical density (ΔOD)) and the density of injected/ejected electrochemical charge (*Q*_d_) necessary to induce a full color change. A quantitative calculation of the coloration efficiency of materials can be done from a plot of the *in situ* optical density at an appropriate wavelength versus the inserted/extracted charge density. A plot of the optical density at 930 nm versus charge density for polymer **P1** is shown in Figure 9B. The coloration efficiency value was extracted as the slope of the line fitted to the linear region of the curve and it was found to be 136.5 cm^2^/C, which is similar to values for other triphenylamine-based polymers obtained via a polycondensation method.^51^

To investigate the stability of the film as a function of time, multiple switching between orange and gray colors was investigated in 10 s intervals (Figure 9C). The 10 s interval was chosen to obtain a high enough color contrast and, due to the fact that the measurement was done using an acetonitrile-based electrolyte, to shorten the duration of the measurement to avoid evaporation of the solvent. It was found that the polymer **P1** was quite stable during ∼275 switching cycles and the transmittance difference dropped by only ∼3.5% from 30.9% to 27.4% but then decreased to 23% when ∼310 switching cycles were carried out .

In the case of polymer **P2** the color change from yellow to gray-blue was observed as a result of oxidation of the triphenylamine group followed by a color change to red as a result of neutralization of the radical cation on the triphenylamine group and reduction of Cu(II) ions to Cu(I) ions (Figure S30). The color changes, although visible by the naked eye, were found to be of very low intensity in terms of differences in both absorbance and transmittance (Figures S30 and S31). Additionally, the polymer was found to exhibit low stability during multiple oxidation/reduction cycles and it delaminated from the surface during 10 oxidation/reduction cycles (Figure S31). This was probably due to the necessity of changes in the conformation of the coordination subunits caused by a change in the coordination preferences of the metallic centers. In the case of polymer **P3** a color change from red to orange has been observed (Figure S32), whereas the polymer **P4** exhibited a color change from yellow to brown after electroreduction (Figure S33). The color changes of polymers **P3** and **P4** were both found to be irreversible.

To investigate the influence of transition-metal ions on the electrochromic properties of the materials, we attempted to prepare thin films of the investigated ligands using the on-substrate polymerization method. It was found that the material obtained by polycondensation of ligand **L** and dialdehyde **4** exhibited reversible oxidation/reduction properties with the anodic and cathodic potentials *E*_pa_ = +1.13 V and *E*_pc_ = +0.72 V, respectively (Figure S34A). In case of the material obtained by polycondensation of ligand **L** and dialdehyde **5**, an irreversible oxidation at *E*_pa_ = +1.99 V was observed (Figure S34B), while the polycondensation of **L** with dialdehyde **6** gave an electrochemically inactive material. The electrochemically induced color change of the thin film was observed only in the case of a material containing a triphenylamine group (Figure S35). The material in its neutral state was yellow with an absorption maxima at 378 nm, and after oxidation of the triphenylamine group, material turned blue, which was concomitant with the appearance of a new, broad absorption band at 700 nm. This indicates that the coordination with metal ions changes the optical properties of the materials.

The surface morphology of the layers of polymers **P1** and **P2** was investigated using scanning electron microscopy (SEM) as well as atomic force electron microscopy (AFM).

**Figure 10:**
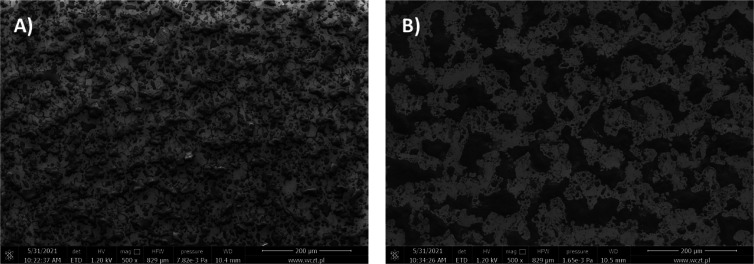
SEM images of layers of polymers **P1** (A) and **P2** (B).

As seen in Figure 10, the polymers do not form a continuous and uniform layer on the surface; instead, the ITO surface is covered by small droplets of the polymers. This is the result of the coating method that was applied. Due to spray-coating of the mixture of monomers onto ITO substrates, small droplets of the mixture of monomers formed and then polymerization occurred within the droplets. During the SEM observation, in order to determine the composition of a certain part of the polymer, an EDX analysis was performed to obtain the elemental composition. EDX spectra of polymers **P1** and **P2** are shown in Figure S36 and S37, and they confirm that polymers **P1** and **P2** contain the transition-metal ions iron and copper, respectively, in the structure, which has also been confirmed by an XPS analysis. Due to the nonuniform nature of the layers, it was difficult to measure their average thickness. The thickness of the islands of polymers was investigated using AFM imaging (Figures S38 and S39). It was found that the thickness of selected islands is as much as 1 μm for polymer **P1** and as much as 2 μm for **P2**.

## Conclusions

In summary, we have prepared the series of polyazomethines **P1**–**P6** containing complexes of transition-metal ions by polycondensation reactions of tetraamines bearing complexes of transition-metal ions with the 2,6-bis(1-hydrazonoethyl)pyridine ligand **L** and different dialdehydes and investigated them with respect to their performance as electrochromic materials. The polymers were obtained by on-substrate polymerization methods, which allowed for the production of polymers as insoluble thin layers on a transparent ITO electrode. Electrochemical and spectroelectrochemical studies reveal that the most interesting properties are exhibited by polymers **P1** and **P2**, obtained by the polycondensation of complexes of both Fe(II) and Cu(II) ions, respectively, with the triphenylamine-based dialdehyde. Polyazomethine **P1** containing Fe(II) ions exhibited a color change from orange to gray, while the Cu(II)-based polyazomethine **P2** changed a color from yellow to blue as a result of electrooxidation and from blue to red after electroreduction of the metallic center. Additionally, polymer **P1** exhibited good electrochromic stability during ∼300 oxidation/reduction cycles, switching times of 9.6 and 6.4 s for the coloring and bleaching steps, respectively, and a coloration efficiency of 136.5 cm^2^/C, which makes it a promising material for electrochromic applications. The research conducted also showed the usefulness of an on-substrate polycondensation reaction for the preparation of thin films of electrochromic metallopolymers.

## Supporting Information Available

Additional figures and a table as described in the text (PDF)

## Accession Codes

CCDC 2075985–2075987 contain the supplementary crystallographic data for this paper. These data can be obtained free of charge via www.ccdc.cam.ac.uk/data_request/cif, or by emailing data_request@ccdc.cam.ac.uk, or by contacting The Cambridge Crystallographic Data Centre, 12 Union Road, Cambridge CB2 1EZ, UK; fax: +44 1223 336033.

## References

[ref1] Liu X.; Rapakousiou A.; Deraedt C.; Ciganda R.; Wang Y.; Ruiz J.; Gu H.; Astruc D. Multiple applications of polymers containing electron-reservoir metal-sandwich complexes. Chem. Commun. 2020, 56, 11374–11385. 10.1039/D0CC04586A. 32990300

[ref2] Yuan M.; Wang F.; Tian Y.-K. Metallo-supramolecular polymers derived from benzothiadiazole-based platinum acetylide complexes for fluorescent security application. RSC Adv. 2018, 8, 40794–40797. 10.1039/C8RA08615J. PMC9091475 35557903

[ref3] Zhang J.; Xu L.; Wong W.-Y. Energy materials based on metal Schiff base complexes. Coord. Chem. Rev. 2018, 355, 180–198. 10.1016/j.ccr.2017.08.007.

[ref4] Cheng K.; Tieke B. Polyiminofluorene with conjugated benzimidazolylpyridine substituent groups: optical properties, ionochromism and coordinative self-assembly into electrochromic films. RSC Adv. 2014, 4, 25079–25088. 10.1039/C4RA03969F.

[ref5] Krieger G.; Tieke B. Coordinative Layer-by-Layer Assembly of Thin Films Based on Metal Ion Complexes of Ligand-Substituted Polystyrene Copolymers and Their Use as Separation Membranes. Macromol. Chem. Phys. 2017, 218, 1700052 10.1002/macp.201700052.

[ref6] Napierała S.; Wałęsa-Chorab M. On-substrate postsynthetic metal ion exchange as a tool for tuning electrochromic properties of materials. Eur. Polym. J. 2020, 140, 110052 10.1016/j.eurpolymj.2020.110052.

[ref7] Maier A.; Rabindranath A. R.; Tieke B. Fast-Switching Electrochromic Films of Zinc Polyiminofluorene-Terpyridine Prepared Upon Coordinative Supramolecular Assembly. Adv. Mater. 2009, 21, 959–963. 10.1002/adma.200802490.

[ref8] Maier A.; Tieke B. Coordinative Layer-by-Layer Assembly of Electrochromic Thin Films based on Metal Ion Complexes of Terpyridine-Substituted Polyaniline Derivatives. J. Phys. Chem. B 2012, 116, 925–934. 10.1021/jp209600d. 22188429

[ref9] Kuai Y.; Li W.; Dong Y.; Wong W.-Y.; Yan S.; Dai Y.; Zhang C. Multi-color electrochromism from coordination nanosheets based on a terpyridine-Fe(ii) complex. Dalton Trans. 2019, 48, 15121–15126. 10.1039/C9DT02980J. 31559982

[ref10] Xu X.; Van Guyse J. F. R.; Jerca V. V.; Hoogenboom R. Metal Ion Selective Self-Assembly of a Ligand Functionalized Polymer into [1 + 1] Macrocyclic and Supramolecular Polymer Structures via Metal–Ligand Coordination. Macromol. Rapid Commun. 2020, 41, 1900305 10.1002/marc.201900305. 31418964

[ref11] Mondal S.; Chandra Santra D.; Ninomiya Y.; Yoshida T.; Higuchi M. Dual-Redox System of Metallo-Supramolecular Polymers for Visible-to-Near-IR Modulable Electrochromism and Durable Device Fabrication. ACS Appl. Mater. Interfaces 2020, 12, 58277–58286. 10.1021/acsami.0c18109. 33326234

[ref12] Leung A. C. W.; Chong J. H.; Patrick B. O.; MacLachlan M. J. Poly(salphenyleneethynylene)s: A New Class of Soluble, Conjugated, Metal-Containing Polymers. Macromolecules 2003, 36, 5051–5054. 10.1021/ma034229l.

[ref13] Puodziukynaite E.; Oberst J. L.; Dyer A. L.; Reynolds J. R. Establishing Dual Electrogenerated Chemiluminescence and Multicolor Electrochromism in Functional Ionic Transition-Metal Complexes. J. Am. Chem. Soc. 2012, 134, 968–978. 10.1021/ja2065297. 22239285

[ref14] Nunes M.; Araújo M.; Fonseca J.; Moura C.; Hillman R.; Freire C. High-Performance Electrochromic Devices Based on Poly[Ni(salen)]-Type Polymer Films. ACS Appl. Mater. Interfaces 2016, 8, 14231–14243. 10.1021/acsami.6b01977. 27175794

[ref15] Ionescu A.; Aiello I.; La Deda M.; Crispini A.; Ghedini M.; De Santo M. P.; Godbert N. Near-IR Electrochromism in Electrodeposited Thin Films of Cyclometalated Complexes. ACS Appl. Mater. Interfaces 2016, 8, 12272–12281. 10.1021/acsami.6b01167. 27115248

[ref16] Nie H. J.; Zhong Y. W. Near-infrared electrochromism in electropolymerized metallopolymeric films of a phen-1,4-diyl-bridged diruthenium complex. Inorg. Chem. 2014, 53, 11316–11322. 10.1021/ic5019967. 25300035

[ref17] Cui B.-B.; Tang J.-H.; Yao J.; Zhong Y.-W. A Molecular Platform for Multistate Near-Infrared Electrochromism and Flip-Flop, Flip-Flap-Flop, and Ternary Memory. Angew. Chem., Int. Ed. 2015, 54, 9192–9197. 10.1002/anie.201504584. 26138863

[ref18] Napierała S.; Kubicki M.; Patroniak V.; Wałęsa-Chorab M. Electropolymerization of [2 × 2] grid-type cobalt(II) complex with thiophene substituted dihydrazone ligand. Electrochim. Acta 2021, 369, 137656 10.1016/j.electacta.2020.137656.

[ref19] Hasanain F.; Wang Z. Y. The synthesis and characterization of near-infrared absorbing, electrochromic polyimides containing a dinuclear ruthenium complex in the polymer mainchain. Dyes Pigm. 2009, 83, 95–101. 10.1016/j.dyepig.2009.03.020.

[ref20] Bandyopadhyay A.; Higuchi M. From metal complexes to metallosupramolecular polymers via polycondensation: Synthesis, structure and electrochromic properties of Co(III)- and Fe(III)-based metallosupramolecular polymers with aromatic azo ligands. Eur. Polym. J. 2013, 49, 1688–1697. 10.1016/j.eurpolymj.2013.03.015.

[ref21] Sicard L.; Navarathne D.; Skalski T.; Skene W. G. On-Substrate Preparation of an Electroactive Conjugated Polyazomethine from Solution-Processable Monomers and its Application in Electrochromic Devices. Adv. Funct. Mater. 2013, 23, 3549–3559. 10.1002/adfm.201203657.

[ref22] Walesa-Chorab M.; Skene W. G. On-substrate polymerization - a versatile approach for preparing conjugated polymers suitable as electrochromes and for metal ion sensing. RSC Adv. 2014, 4, 19053–19060. 10.1039/c4ra00721b.

[ref23] Mulholland M. E.; Navarathne D.; Petrus M. L.; Dingemans T. J.; Skene W. G. Correlating on-substrate prepared electrochromes with their solution processed counterparts – towards validating polyazomethines as electrochromes in functioning devices. J. Mater. Chem. C 2014, 2, 9099–9108. 10.1039/C4TC01003E.

[ref24] Wałęsa-Chorab M.; Banasz R.; Kubicki M.; Patroniak V. Dipyrromethane functionalized monomers as precursors of electrochromic polymers. Electrochim. Acta 2017, 258, 571–581. 10.1016/j.electacta.2017.11.100.

[ref25] Williams D. B.; Lawton M. Drying of organic solvents: quantitative evaluation of the efficiency of several desiccants. J. Org. Chem. 2010, 75, 8351–8354. 10.1021/jo101589h. 20945830

[ref26] CrysAlisPro 1.171.40.53; Rigaku Oxford Diffraction: 2019.

[ref27] Sheldrick G. SHELXT - Integrated space-group and crystal-structure determination. Acta Crystallogr., Sect. A: Found. Adv. 2015, 71, 3–8. 10.1107/S2053273314026370. 25537383 PMC4283466

[ref28] Sheldrick G. Crystal structure refinement with SHELXL. Acta Crystallogr., Sect. C: Struct. Chem. 2015, 71, 3–8. 10.1107/S2053229614024218. 25567568 PMC4294323

[ref29] Shee N. K.; Drew M. G. B.; Datta D. Tuning of the lowest excited states in mixed ruthenium(ii) polypyridyl complexes having RuN6 cores by the conformation of the ancillary ligand. Emission from a 3ligand-to-ligand-charge-transfer state. New J. Chem. 2016, 40, 5002–5009. 10.1039/C5NJ03329B.

[ref30] Chen W.-C.; Wu G.-F.; Yuan Y.; Wei H.-X.; Wong F.-L.; Tong Q.-X.; Lee C.-S. A meta-molecular tailoring strategy towards an efficient violet-blue organic electroluminescent material. RSC Adv. 2015, 5, 18067–18074. 10.1039/C4RA16954A.

[ref31] Shee N. K.; Dutta S.; Drew M. G. B.; Datta D. Bis complexes of zinc(II), cadmium(II) and mercury(II) with a potentially pentadentate N-donor ligand. Lewis acidity versus coordination tendency. Inorg. Chim. Acta 2013, 398, 132–135. 10.1016/j.ica.2012.12.024.

[ref32] Radecka-Paryzek W.; Gdaniec M. The preparation, spectral and X-ray crystallographic characterization of 2,6-diacetylpyridinedihydrazone complex with lead(II) nitrate. Polyhedron 1997, 16, 3681–3686. 10.1016/S0277-5387(97)00099-5.

[ref33] Anacona J. R.; Rangel V.; Loroño M.; Camus J. Tetradentate metal complexes derived from cephalexin and 2,6-diacetylpyridine bis(hydrazone): Synthesis, characterization and antibacterial activity. Spectrochim. Acta, Part A 2015, 149, 23–29. 10.1016/j.saa.2015.04.054. 25942081

[ref34] Gup R.; Gökçe C.; Dilek N. Synthesis, structural characterization and DNA interaction of zinc complex from 2,6-diacetylpyridine dihydrazone and {4-[(2E)-2-(hydroxyimino)acetyl]phenoxy} acetic acid. J. Photochem. Photobiol., B 2015, 144, 42–50. 10.1016/j.jphotobiol.2015.02.002. 25704313

[ref35] Liu Z.; Ou J.; Wang H.; You X.; Ye M. Synthesis and Characterization of Hydrazide-Linked and Amide-Linked Organic Polymers. ACS Appl. Mater. Interfaces 2016, 8, 32060–32067. 10.1021/acsami.6b11572. 27809468

[ref36] Coogan N. T.; Chimes M. A.; Raftery J.; Mocilac P.; Denecke M. A. Regioselective Synthesis of V-Shaped Bistriazinyl-phenanthrolines. J. Org. Chem. 2015, 80, 8684–8693. 10.1021/acs.joc.5b01380. 26237435

[ref37] Zare N.; Zabardasti A. A new nano-sized mononuclear Cu (II) complex with N,N-donor Schiff base ligands: sonochemical synthesis, characterization, molecular modeling and biological activity. Appl. Organomet. Chem. 2019, 33, e4687 10.1002/aoc.4687.

[ref38] Eguchi M.; Momotake M.; Inoue F.; Oshima T.; Maeda K.; Higuchi M. Inert Layered Silicate Improves the Electrochemical Responses of a Metal Complex Polymer. ACS Appl. Mater. Interfaces 2017, 9, 35498–35503. 10.1021/acsami.7b13311. 28933528

[ref39] Duchanois T.; Etienne T.; Cebrián C.; Liu L.; Monari A.; Beley M.; Assfeld X.; Haacke S.; Gros P. C. An Iron-Based Photosensitizer with Extended Excited-State Lifetime: Photophysical and Photovoltaic Properties. Eur. J. Inorg. Chem. 2015, 2015, 2469–2477. 10.1002/ejic.201500142.

[ref40] Vukadinovic Y.; Burkhardt L.; Papcke A.; Miletic A.; Fritsch L.; Altenburger B.; Schoch R.; Neuba A.; Lochbrunner S.; Bauer M. When Donors Turn into Acceptors: Ground and Excited State Properties of Fe(II) Complexes with Amine-Substituted Tridentate Bis-imidazole-2-ylidene Pyridine Ligands. Inorg. Chem. 2020, 59, 8762–8774. 10.1021/acs.inorgchem.0c00393. 32530276

[ref41] Vila N.; Walcarius A. Bis(terpyridine) Iron(II) Functionalized Vertically-Oriented Nanostructured Silica Films: Toward Electrochromic Materials. Front. Chem. 2020, 8, 830 10.3389/fchem.2020.00830. 33094099 PMC7523427

[ref42] Bocian A.; Napierała S.; Gorczyński A.; Kubicki M.; Wałęsa-Chorab M.; Patroniak V. The first example of an asymmetrical μ-oxo bridged dinuclear iron complex with a terpyridine ligand. New J. Chem. 2019, 43, 12650–12656. 10.1039/C9NJ02413A.

[ref43] Fontanesi C.; Como E. D.; Vanossi D.; Montecchi M.; Cannio M.; Mondal P. C.; Giurlani W.; Innocenti M.; Pasquali L. Redox-Active Ferrocene grafted on H-Terminated Si(111): Electrochemical Characterization of the Charge Transport Mechanism and Dynamics. Sci. Rep. 2019, 9, 8735 10.1038/s41598-019-45448-w. 31217551 PMC6584626

[ref44] Eckermann A. L.; Feld D. J.; Shaw J. A.; Meade T. J. Electrochemistry of redox-active self-assembled monolayers. Coord. Chem. Rev. 2010, 254, 1769–1802. 10.1016/j.ccr.2009.12.023. 20563297 PMC2885823

[ref45] Hrbac J.; Storch J.; Halouzka V.; Cirkva V.; Matejka P.; Vacek J. Immobilization of helicene onto carbon substrates through electropolymerization of [7]helicenyl-thiophene. RSC Adv. 2014, 4, 46102–46105. 10.1039/C4RA06283C.

[ref46] Kim H.-J.; Piao M.-H.; Choi S.-H.; Shin C.-H.; Lee Y.-T. Development of Amperometric Hydrogen Peroxide Sensor Based on Horseradish Peroxidase-Immobilized Poly(Thiophene-co-EpoxyThiophene). Sensors 2008, 8, 4110–4118. 10.3390/s8074110. 27879925 PMC3697164

[ref47] Chábera P.; Liu Y.; Prakash O.; Thyrhaug E.; Nahhas A. E.; Honarfar A.; Essén S.; Fredin L. A.; Harlang T. C. B.; Kjær K. S.; Handrup K.; Ericson F.; Tatsuno H.; Morgan K.; Schnadt J.; Häggström L.; Ericsson T.; Sobkowiak A.; Lidin S.; Huang P.; Styring S.; Uhlig J.; Bendix J.; Lomoth R.; Sundström V.; Persson P.; Wärnmark K. A low-spin Fe(iii) complex with 100-ps ligand-to-metal charge transfer photoluminescence. Nature 2017, 543, 695–699. 10.1038/nature21430. 28358064

[ref48] Banasz R.; Kubicki M.; Walesa-Chorab M. Yellow-to-brown and yellow-to-green electrochromic devices based on complexes of transition metal ions with a triphenylamine-based ligand. Dalton Trans. 2020, 49, 15041–15053. 10.1039/D0DT03232H. 33103702

[ref49] Wałęsa-Chorab M.; Tremblay M.-H.; Skene W. G. Hydrogen-Bond and Supramolecular-Contact Mediated Fluorescence Enhancement of Electrochromic Azomethines. Chem. - Eur. J. 2016, 22, 11382–11393. 10.1002/chem.201600859. 27388588

[ref50] Wałęsa-Chorab M.; Skene W. G. Investigation of an electroactive immobilized azomethine for potential electrochromic use. Sol. Energy Mater. Sol. Cells 2019, 200, 109977 10.1016/j.solmat.2019.109977.

[ref51] Hsiao S.-H.; Liao W.-K.; Liou G.-S. A comparative study of redox-active, ambipolar electrochromic triphenylamine-based polyimides prepared by electrochemical polymerization and conventional polycondensation methods. Polym. Chem. 2018, 9, 236–248. 10.1039/C7PY01897E.

