# Supplementary material for: Toward Electrochromic Metallopolymers: Synthesis and Properties of Polyazomethines Based on Complexes of Transition-Metal Ions
Source: Inorg Chem. 2021 Aug 16;60(18):14011–21. doi: 10.1021/acs.inorgchem.1c01249 (PMC8456411; doi:10.1021/acs.inorgchem.1c01249)
Supplement: Supplementary file 1 — ic1c01249_si_001.pdf [file ic1c01249_si_001.pdf]

Supporting Information

Towards electrochromic metallopolymers: synthesis and properties of polyazomethines based on complexes of transition metal ions

Sergiusz Napierała, Maciej Kubicki, Monika Wałęsa-Chorab*

Faculty of Chemistry, Adam Mickiewicz University in Poznań, Uniwersytetu Poznańskiego 8, 61-614 Poznań, Poland,

Corresponding author e-mail: mchorab@amu.edu.pl

Table S 1. Crystal data, data collection and structure refinement. 4

Figure S 1. FT-IR spectra of ligand **L**. 5
Figure S 2. FT-IR spectra of complex **1**. 5
Figure S 3. FT-IR of complex **2**. 5
Figure S 4. FT-IR spectra of complex **3**. 6
Figure S 5. TGA analysis of ligand **L**. 6
Figure S 6. TGA analysis of complex **1**. 7
Figure S 7. TGA analysis of complex **2**. 7
Figure S 8. TGA analysis of complex **3**. 8
Figure S 9. TGA analysis of polymer **P1**. 8
Figure S 10. TGA analysis of polymer **P2**. 9
Figure S 11. TGA analysis of polymer **P3**. 9
Figure S 12. TGA analysis of polymer **P4**. 10
Figure S 13. TGA analysis of polymer **P5**. 10
Figure S 14. TGA analysis of polymer **P6**. 11
Figure S 15. FT-IR spectra of polymer **P1**. 11
Figure S 16. FT-IR spectra of polymer **P2**. 11
Figure S 17. FT-IR spectra of polymer **P3**. 12
Figure S 18. FT-IR spectra of polymer **P4**. 12
Figure S 19. FT-IR spectra of polymer **P5**. 12
Figure S 20. FT-IR spectra of polymer **P6**. 13
Figure S 21. XPS survey spectra of polymers **P1** (red), **P3** (green), **P5** (blue) and complex **1** (black). 13
Figure S 22. XPS survey spectra of polymers **P2** (red), **P4** (green), **P6** (blue) and complex 2 (black). 13
Figure S 23. X-ray photoelectron spectroscopy patterns of Fe 2p core levels of complex **1** and polymer **P1** (left), and Cu 2p core levels of complex **2** and polymer **P2** (right). 14
Figure S 24. UV-Vis spectra of ligand **L** in the range 300-800 nm. 14
Figure S 25. The anodic part of the cyclic voltammogram of polymer **P1** and fit peaks. 15
Figure S 26. CV profiles of polymer **P1** at different scan rates.. 15
Figure S 27. Linear dependence of the peak current on the scan rate of the polymer **P1**. 16
Figure S 28. Cyclic voltammogram of A) polymer **P3** and B) polymer **P4** measured in anhydrous and deaerated 0.1 M solution of $TBAPF_6$ in acetonitrile. 16
Figure S 29. Electrochromic switching of the polymer **P1** immobilized onto ITO glass slide electrode measured in anhydrous and deaerated 0.1M solution of $TBAPF_6$ in acetonitrile by switching between +1.5V and -0.1V in 30s intervals monitored at 930 nm. 17
Figure S 30. UV-vis spectra of polymer **P2** measured in anhydrous and deaerated 0.1M $TBAPF_6$ in acetonitrile as supporting electrolyte by applying 0 (■), +1.2 V (●) and -0.6 V (▲) potentials versus Ag/Ag^+ reference electrode held for 30 sec per potential. Insert: photographs of the original (left), electrooxidized (middle) and electroreduced (right) **P2**. 17
Figure S 31. Electrochromic stability of the polymer **P2** immobilized onto ITO glass slide electrode measured in anhydrous and deaerated 0.1M solution of $TBAPF_6$ in acetonitrile by switching between +1.2V and -0.5V in 10s intervals monitored at 750 nm. 18
Figure S 32. UV-Vis spectra of polymer **P3** in its neutral (black) and electrochemically oxidized (red) states. Insert: photographs of the original (left), electrooxidized (right) **P3**. 18
Figure S 33. UV-Vis spectra of polymer **P4** in its neutral (black) and electrochemically reduced (red) states. Insert: photographs of the original (left), electroreduced (right) **P4**.. 19

Figure S 34. Cyclic voltammogram thin films obtained by on-substrate polycondensation of **L** with A) dialdehyde **4** and B) dialdehyde **5** measured in anhydrous and deaerated 0.1 M solution of $TBAPF_6$ in acetonitrile. 19
Figure S 35. UV-vis spectra of thin film obtained by polycondensation of **L** with dialdehyde **4** measured in anhydrous and deaerated 0.1M $TBAPF_6$ in acetonitrile as supporting electrolyte by applying 0 (black) and +1.2 V (red) potentials versus Ag/Ag^+ reference electrode held for 30 sec per potential. 20
Figure S 36. Scanning Electron Microscopy (SEM) and EDX analysis of polymer **P1**. 20
Figure S 37. Scanning Electron Microscopy (SEM) and EDX analysis of polymer **P2**. 20
Figure S 38. AFM micrographs of polymer **P1** on ITO electrode. AFM cross-section profiles were measured at a marked places (left). AFM cross-section profiles of **P1** measured at marked places..... 21
Figure S 39. AFM micrographs of polymer **P2** on ITO electrode. AFM cross-section profiles were measured at a marked places (left). AFM cross-section profiles of **P2** measured at marked places..... 21
Figure S 40. ^{1}H NMR of ligand **L** in d_6-DMSO. 22
Figure S 41. ^{13}C NMR spectra of ligand **L**. 22
Figure S 42. ^{1}H NMR of dialdehyde **6** in $CDCl_3$. 23
Figure S 43. ^{13}C NMR of dialdehyde **6** in $CDCl_3$. 23
Figure S 44. HR-ESI-MS of ligand **L**. 24
Figure S 45. HR-ESI-MS spectra of complex **1**. 25
Figure S 46. HR-ESI-MS spectra of complex **2**. 25
Figure S 47. HR-ESI-MS spectra of complex **3**. 25

Table S 1. Crystal data, data collection and structure refinement.

Compound	**1**	**2**	**3**
Formula	$C_{18}H_{26}FeN_{10}^{2+}\cdot 2BF_4^{-}\cdot C_2H_3N$	$C_{18}H_{26}CuN_{10}^{2+}\cdot 2CF_3O_3S^{-}$	$C_{18}H_{26}CuN_{10}^{2+}\cdot 2BF_4^{-}\cdot 2C_2H_3N$
Formula weight	653.01	744.17	701.75
Crystal system	monoclinic	monoclinic	triclinic
Space group	Pn	$P2_1/n$	P-1
a(Å)	8.8502(3)	8.1071(2)	7.2436(4)
b(Å)	26.8633(7)	29.7026(9)	10.3290(5)
c(Å)	11.9038(4)	14.6179(4)	21.2449(9)
α(°)	90	90	79.081(4)
β(°)	102.099(3)	96.126(2)	82.710(4)
γ(°)	90	90	86.005(4)
V(Å^3)	2767.21(15)	3499.92(17)	1546.43(13)
Z	4	4	2
D_x(g cm^{-3})	1.567	1.412	1.507
F(000)	1336	1516	718
μ(mm^{-1})	0.633	0.822	0.791
Reflections:			
collected	20758	15952	11404
unique (R_{int})	10041 (0.0394)	7473 (0.0204)	6550 (0.0178)
with I>2σ(I)	8432	6401	5730
R(F) [I>2σ(I)]	0.0545	0.0455	0.0362
wR(F^2) [I>2σ(I)]	0.1194	0.1124	0.0802
R(F) [all data]	0.0704	0.0541	0.0436
wR(F^2) [all data]	0.1283	0.1177	0.0836
Goodness of fit	1.087	1.005	1.036
max/min Δρ (e·Å^{-3})	1.55/-0.65	0.86/-0.72	0.41/-0.39
CCDC deposition number	2075985	2075986	2075987

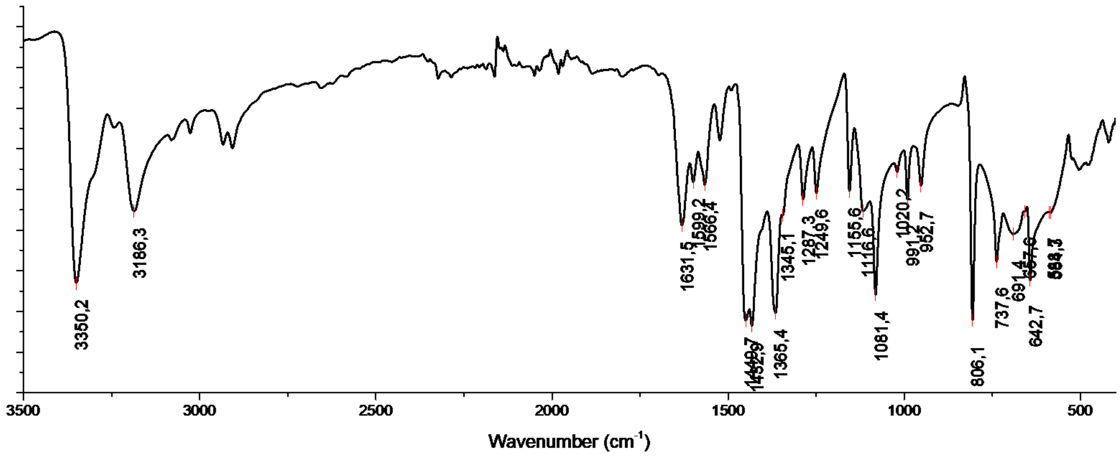

Figure S 1. FT-IR spectra of ligand **L**.

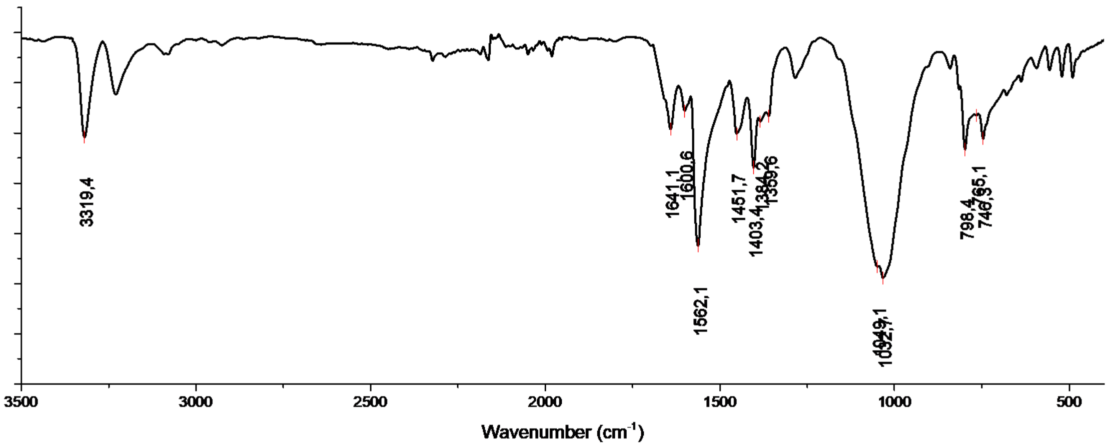

Figure S 2. FT-IR spectra of complex **1**.

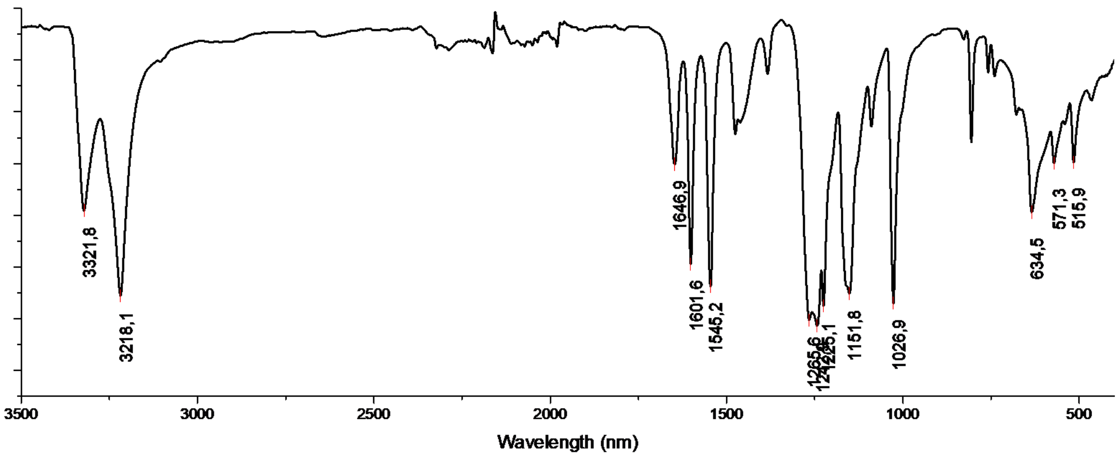

Figure S 3. FT-IR of complex **2**.

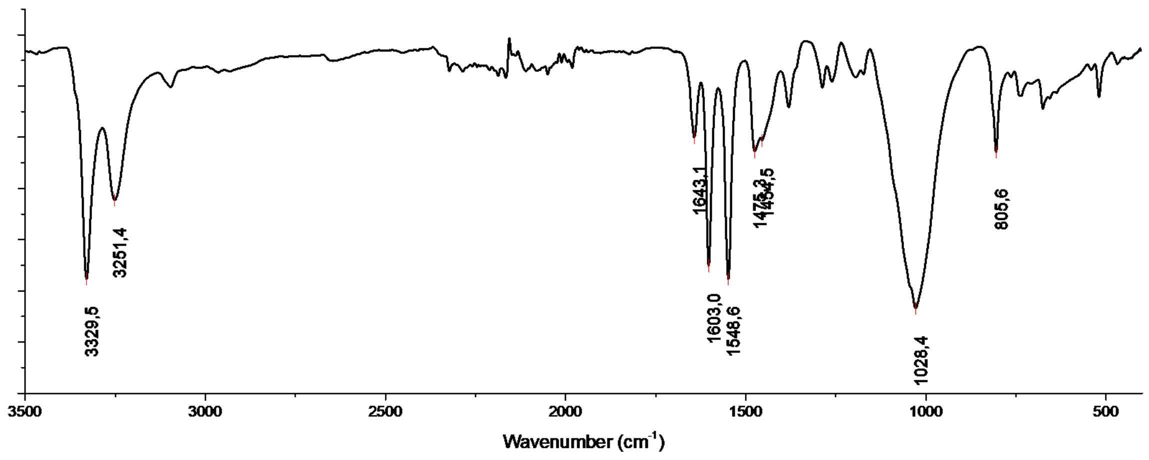

Figure S 4. FT-IR spectra of complex **3**.

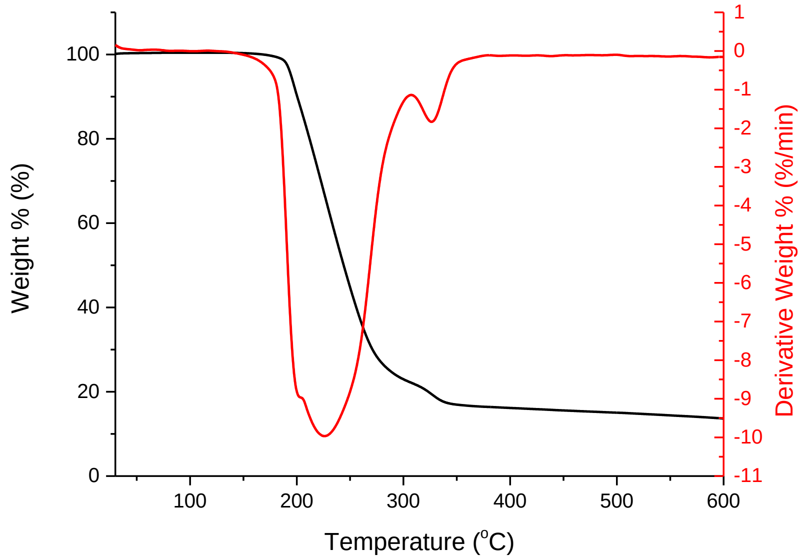

Figure S 5. TGA analysis of ligand **L**.

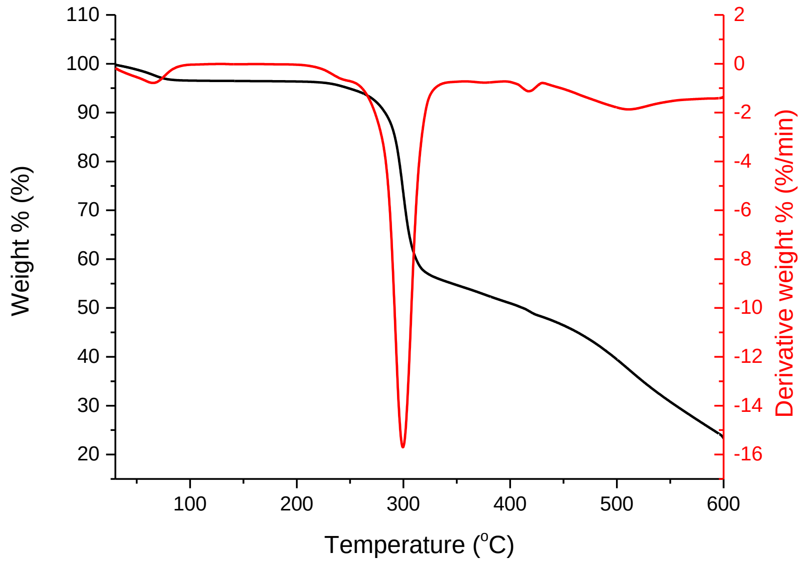

Figure S 6. TGA analysis of complex **1**.

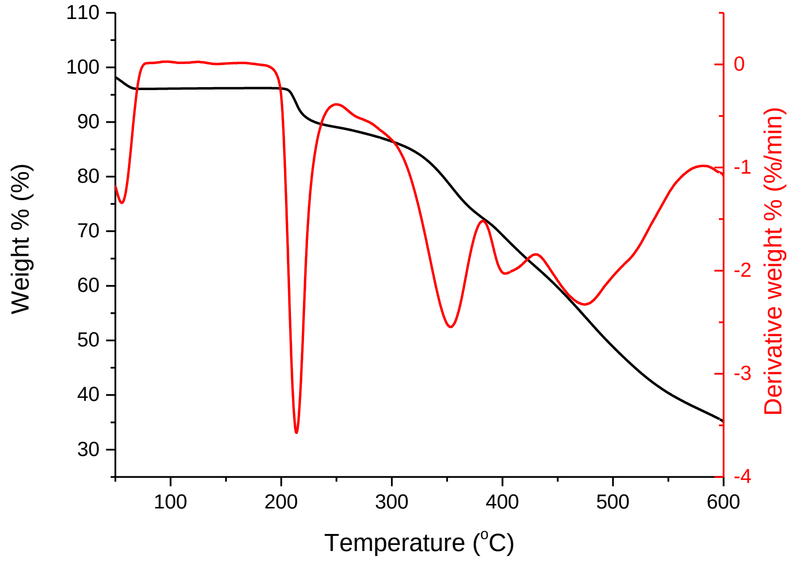

Figure S 7. TGA analysis of complex **2**.

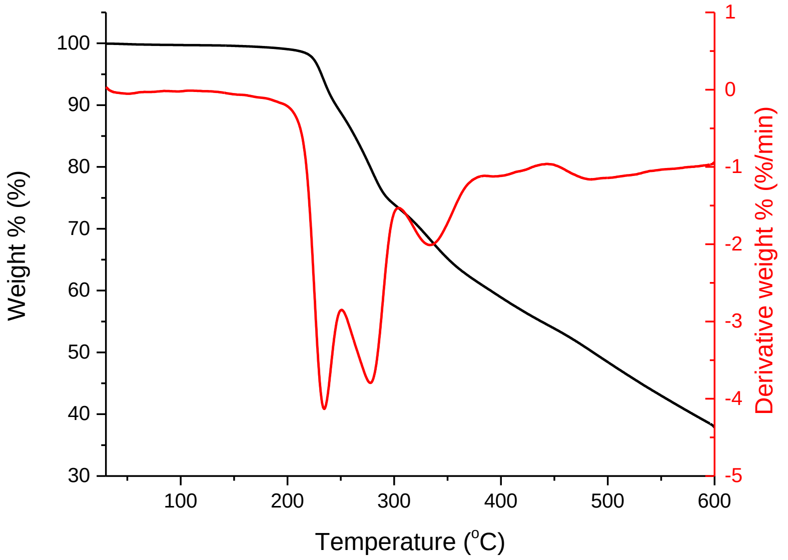

Figure S 8. TGA analysis of complex **3**.

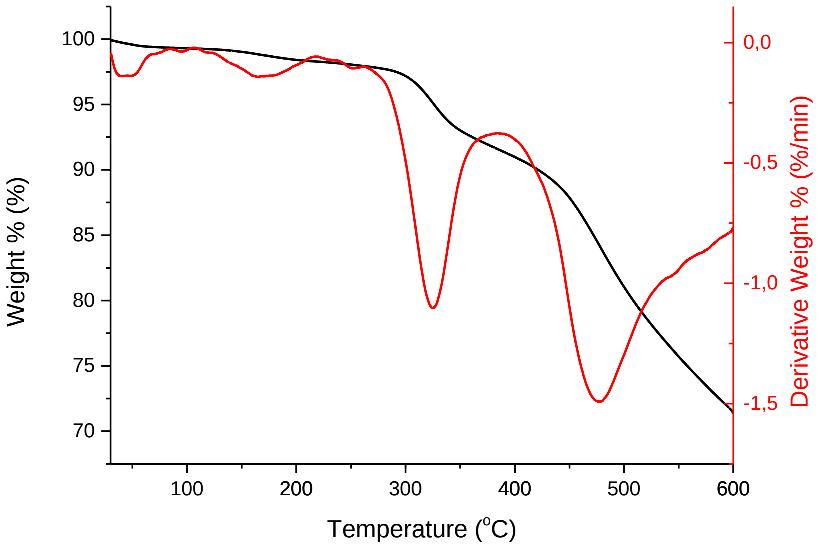

Figure S 9. TGA analysis of polymer **P1**.

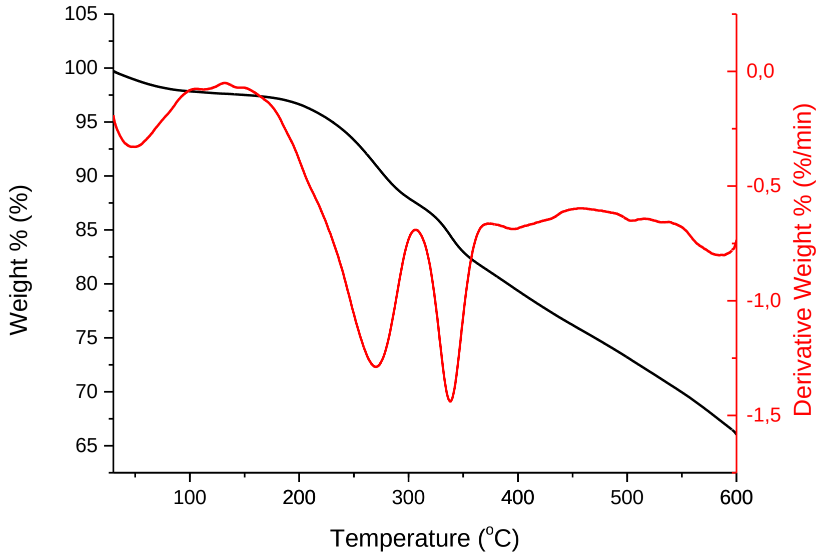

Figure S 10. TGA analysis of polymer **P2**.

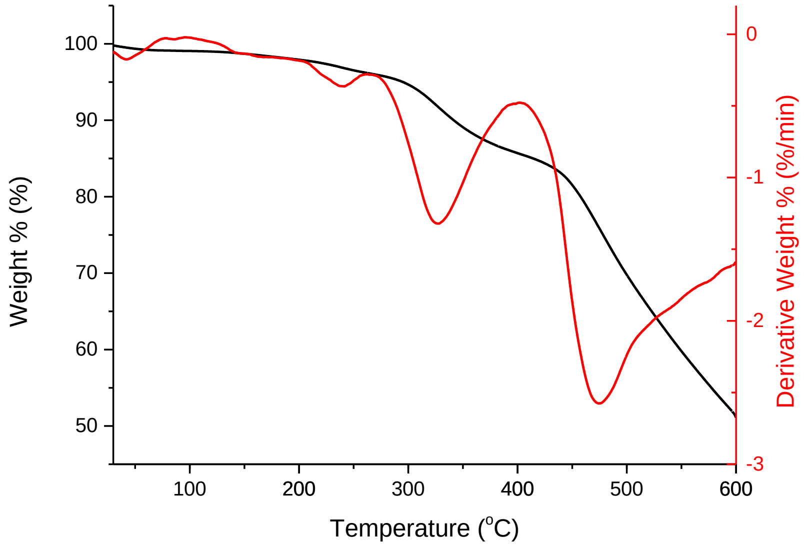

Figure S 11. TGA analysis of polymer **P3**.

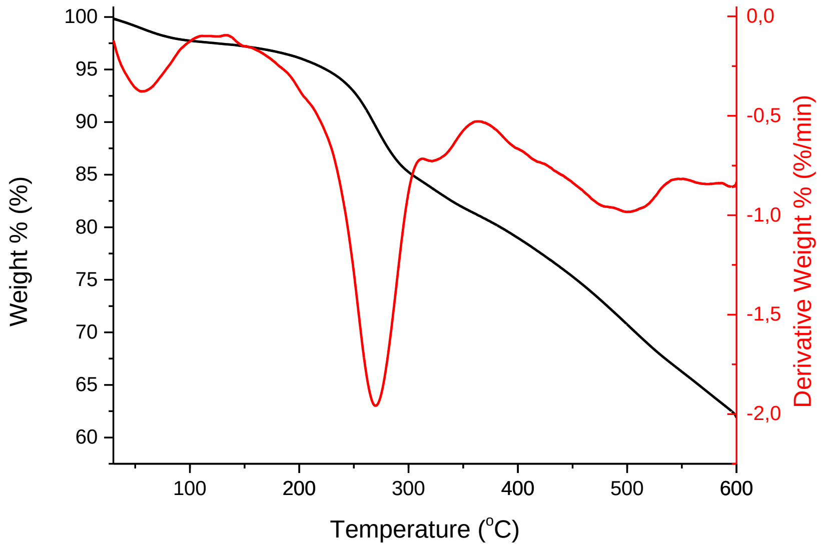

Figure S 12. TGA analysis of polymer **P4**.

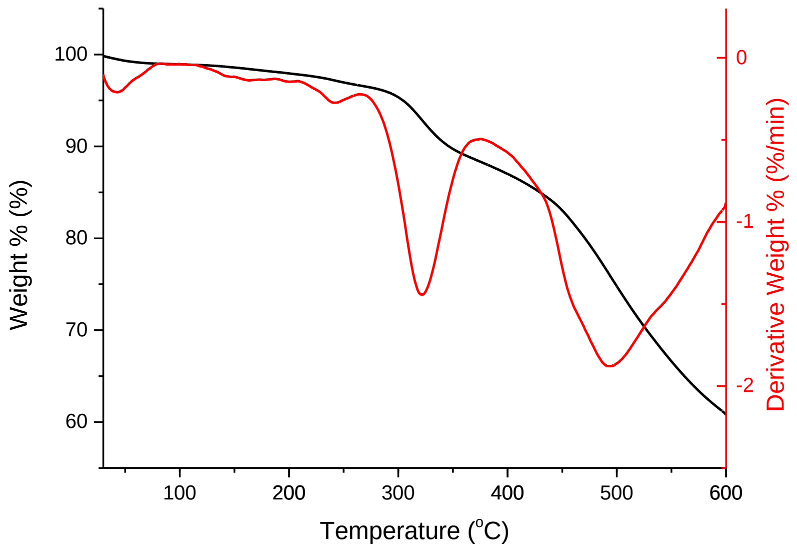

Figure S 13. TGA analysis of polymer **P5**.

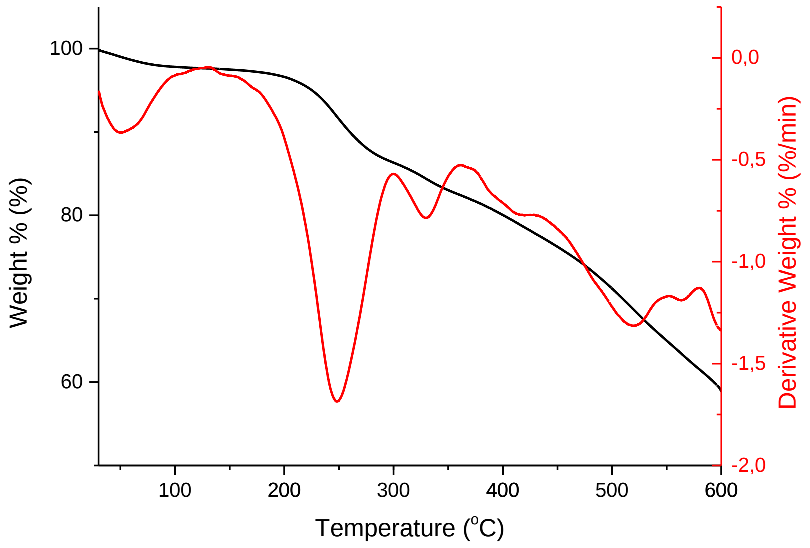

Figure S 14. TGA analysis of polymer **P6**.

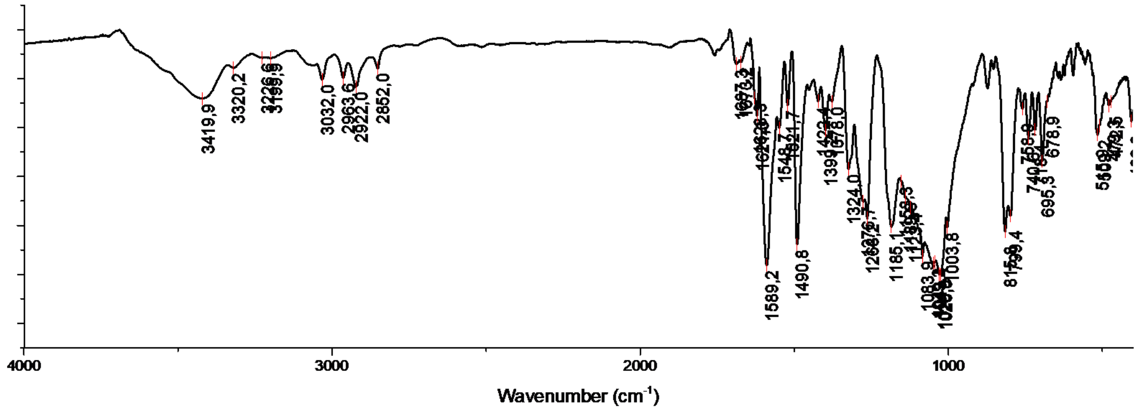

Figure S 15. FT-IR spectra of polymer **P1**.

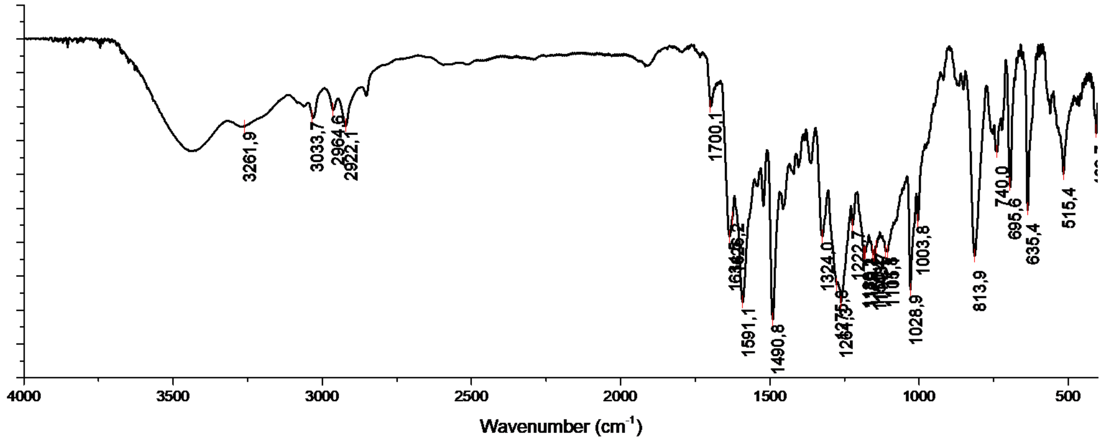

Figure S 16. FT-IR spectra of polymer **P2**.

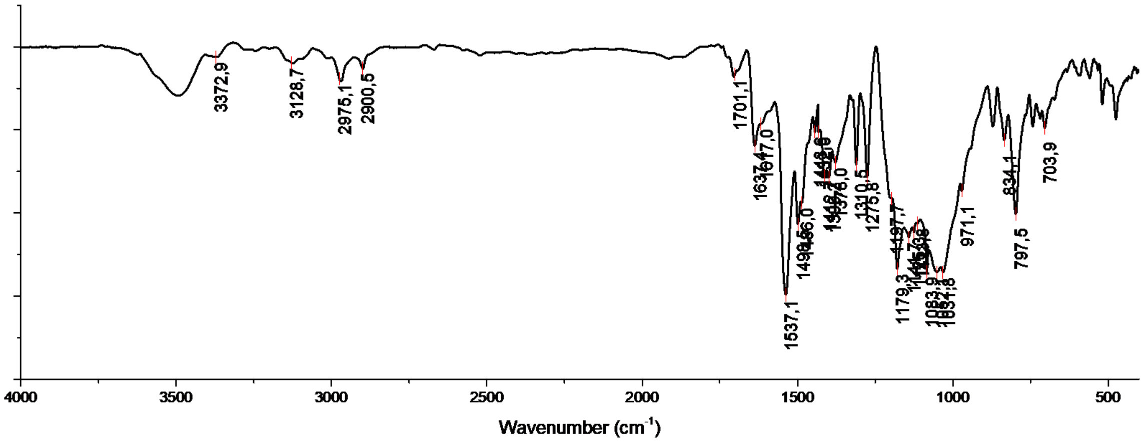

Figure S 17. FT-IR spectra of polymer **P3**.

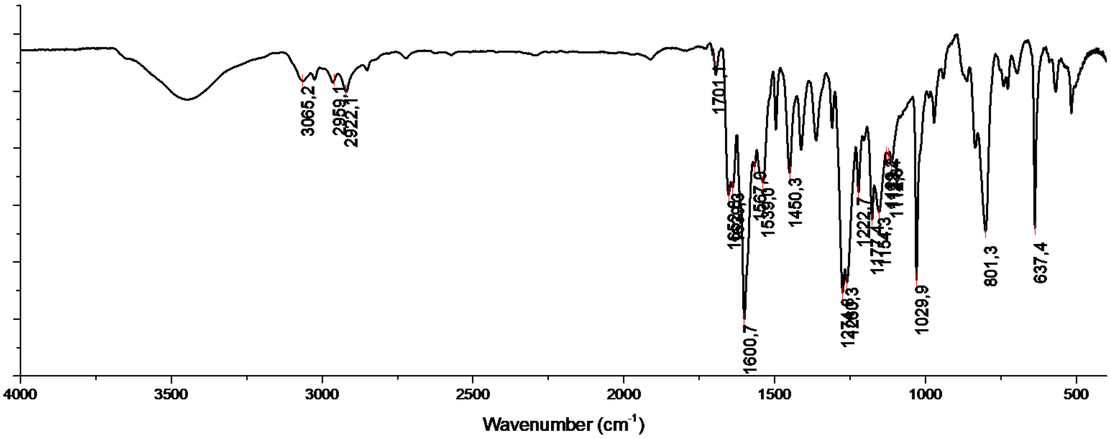

Figure S 18. FT-IR spectra of polymer **P4**.

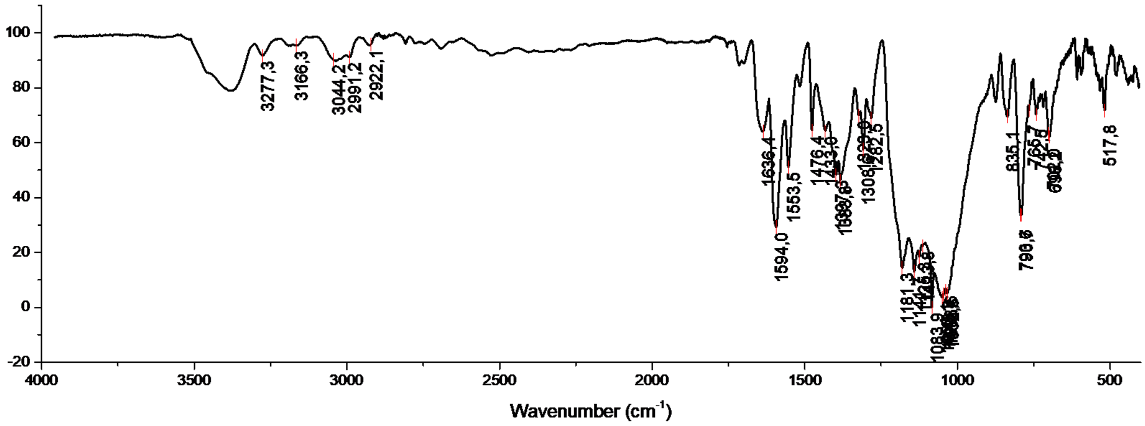

Figure S 19. FT-IR spectra of polymer **P5**.

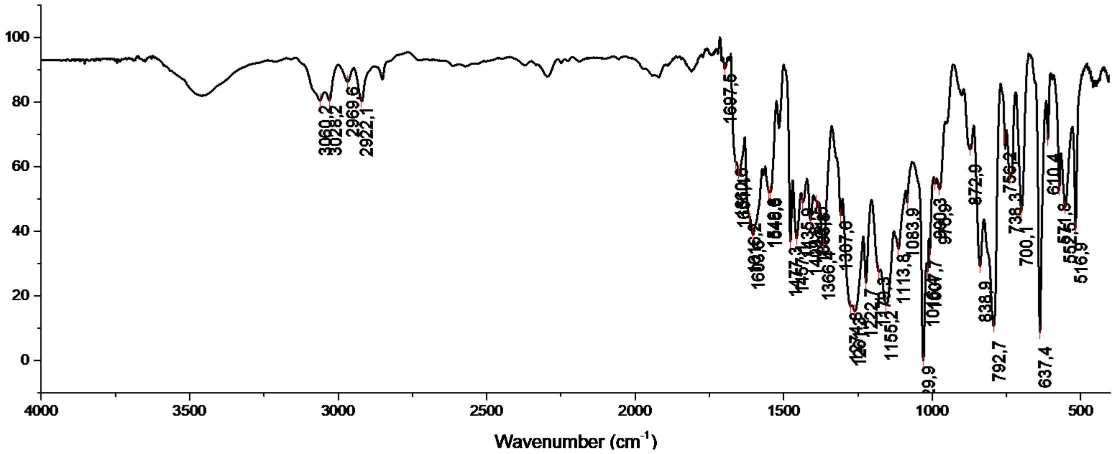

Figure S 20. FT-IR spectra of polymer **P6**.

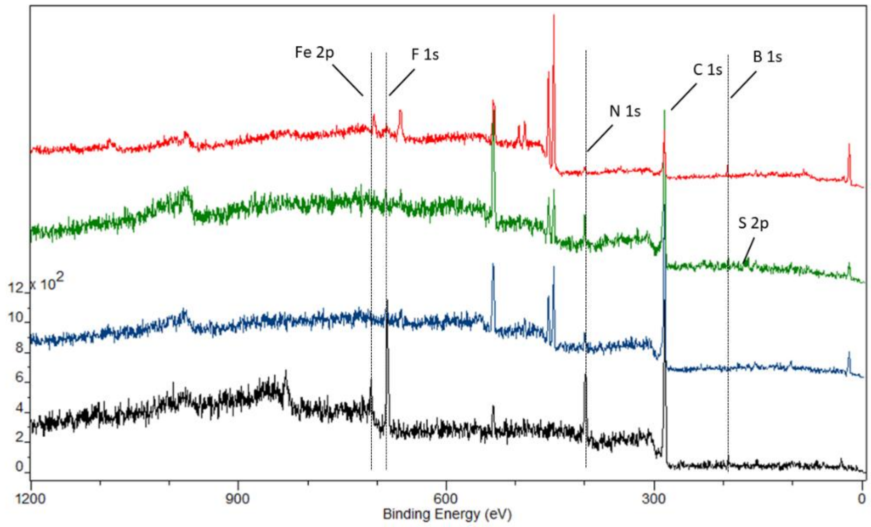

Figure S 21. XPS survey spectra of polymers **P1** (red), **P3** (green), **P5** (blue) and complex **1** (black).

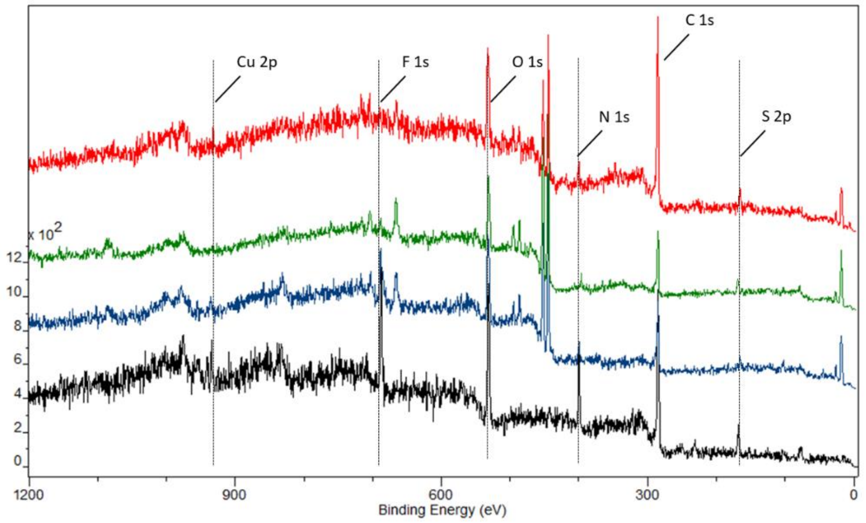

Figure S 22. XPS survey spectra of polymers **P2** (red), **P4** (green), **P6** (blue) and complex 2 (black).

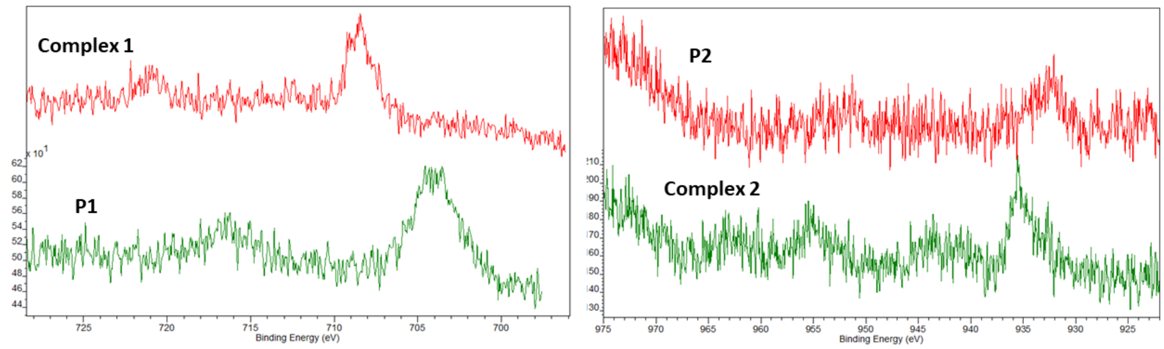

Figure S 23. X-ray photoelectron spectroscopy patterns of Fe 2p core levels of complex **1** and polymer **P1** (left), and Cu 2p core levels of complex **2** and polymer **P2** (right).

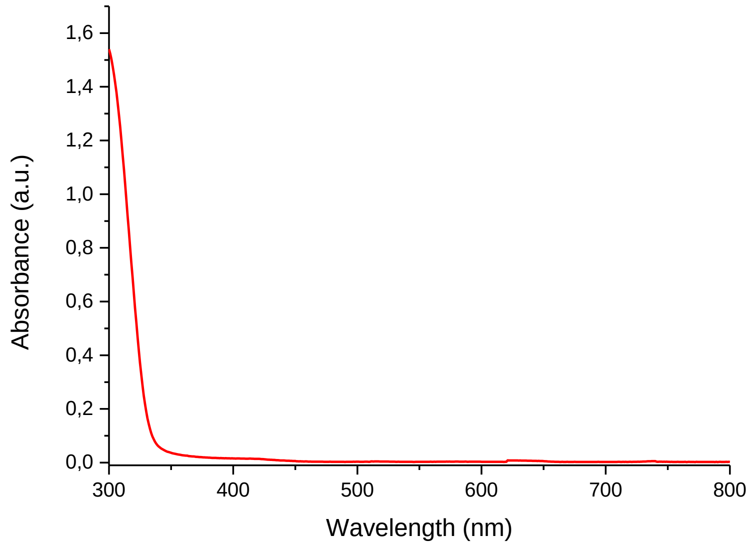

Figure S 24. UV-Vis spectra of ligand **L** in the range 300-800 nm.

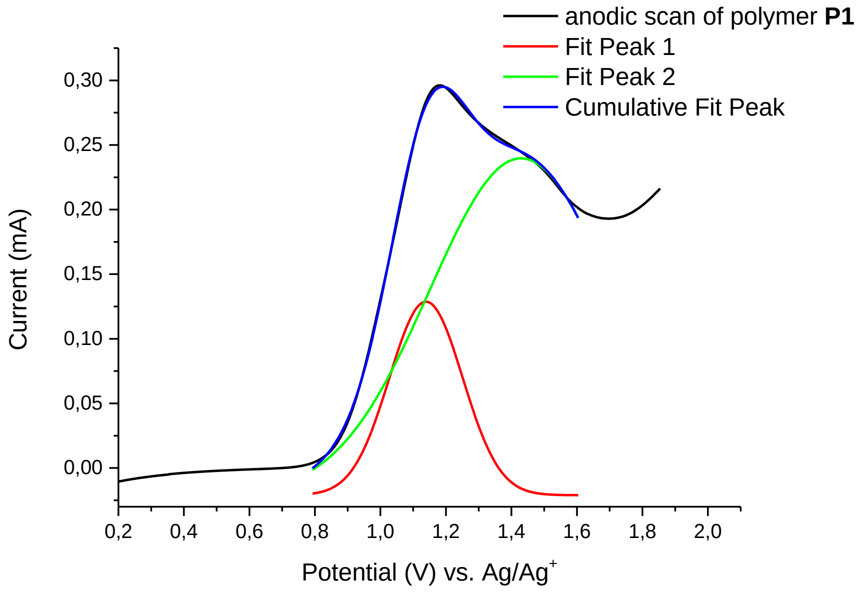

Figure S 25. The anodic part of the cyclic voltammogram of polymer **P1** and fit peaks.

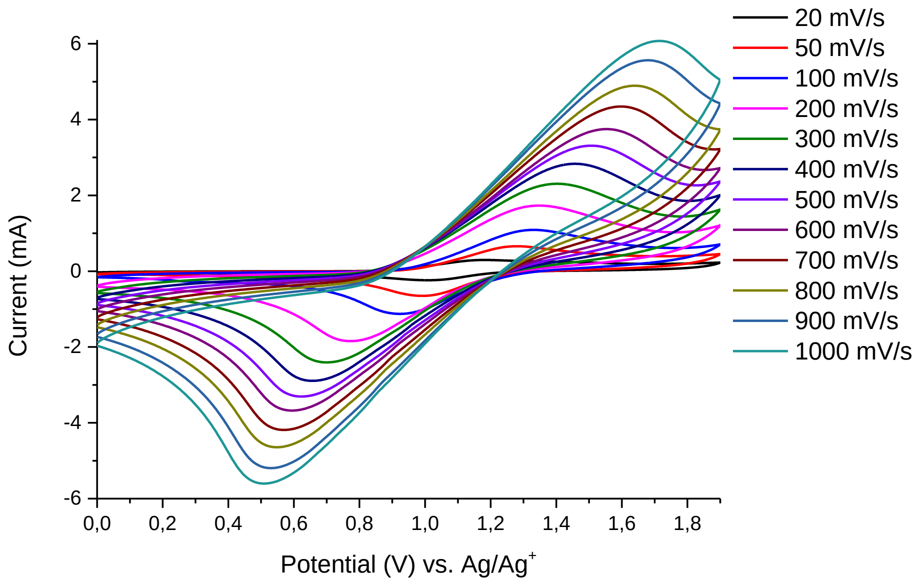

Figure S 26. CV profiles of polymer **P1** at different scan rates.

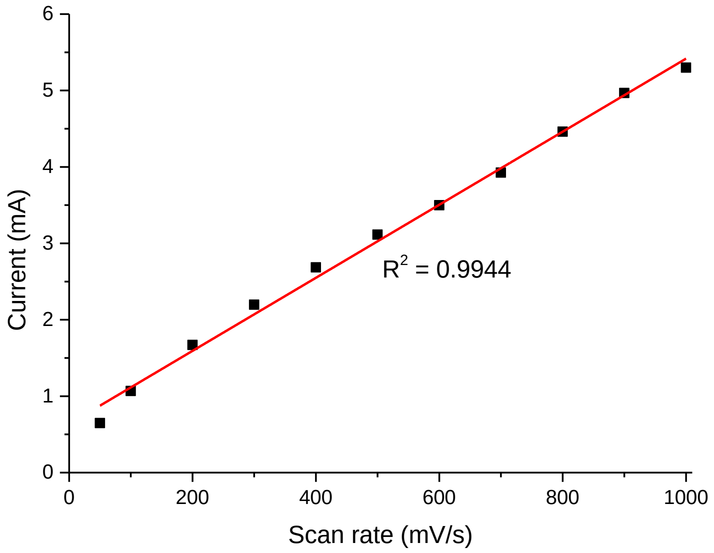

Figure S 27. Linear dependence of the peak current on the scan rate of the polymer **P1**.

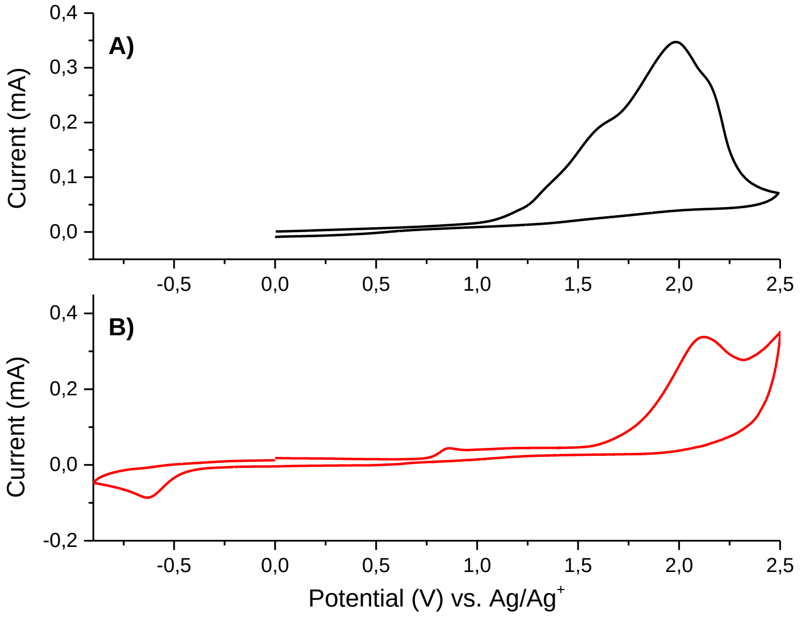

Figure S 28. Cyclic voltammogram of A) polymer **P3** and B) polymer **P4** measured in anhydrous and deaerated 0.1 M solution of $TBAPF_6$ in acetonitrile.

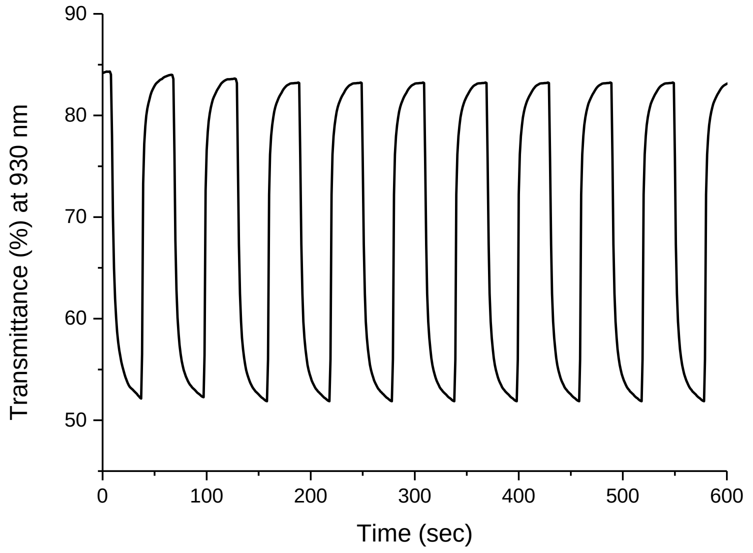

Figure S 29. Electrochromic switching of the polymer **P1** immobilized onto ITO glass slide electrode measured in anhydrous and deaerated 0.1M solution of $TBAPF_6$ in acetonitrile by switching between +1.5V and -0.1V in 30s intervals monitored at 930 nm.

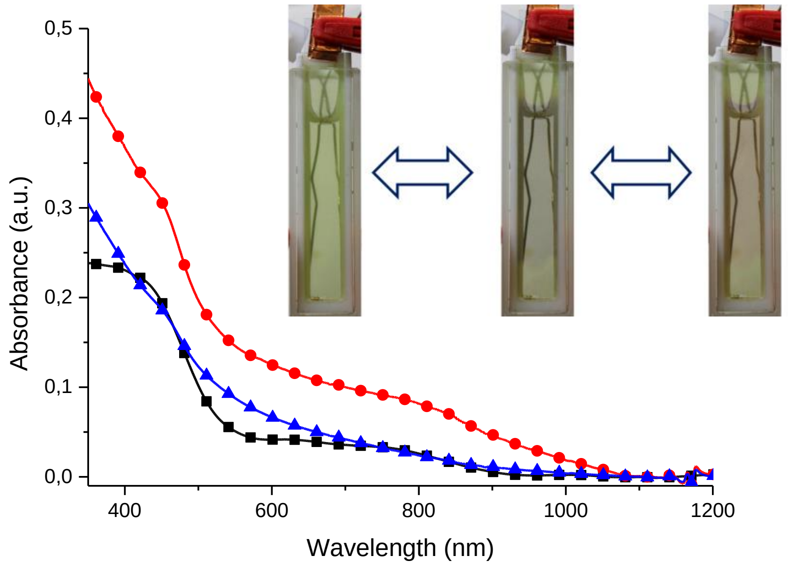

Figure S 30. UV-vis spectra of polymer **P2** measured in anhydrous and deaerated 0.1M $TBAPF_6$ in acetonitrile as supporting electrolyte by applying 0 (■), +1.2 V (●) and -0.6 V (▲) potentials versus Ag/Ag^+ reference electrode held for 30 sec per potential. Insert: photographs of the original (left), electrooxidized (middle) and electroreduced (right) **P2**.

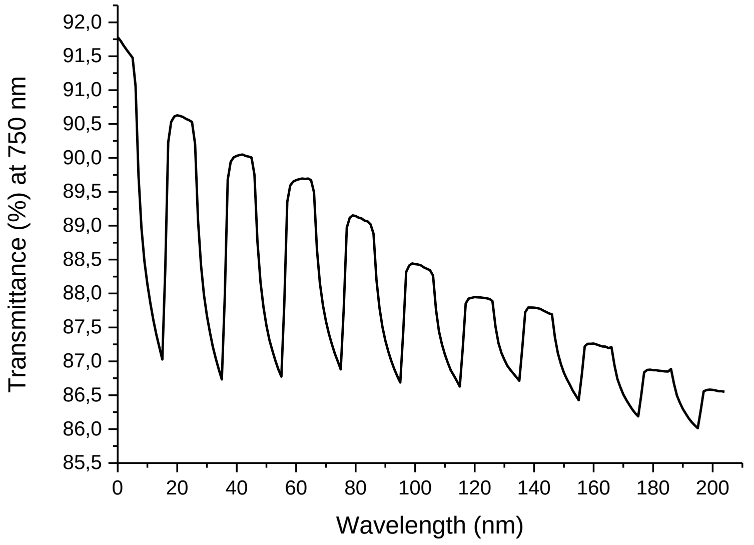

Figure S 31. Electrochromic stability of the polymer **P2** immobilized onto ITO glass slide electrode measured in anhydrous and deaerated 0.1M solution of $TBAPF_6$ in acetonitrile by switching between +1.2V and -0.5V in 10s intervals monitored at 750 nm.

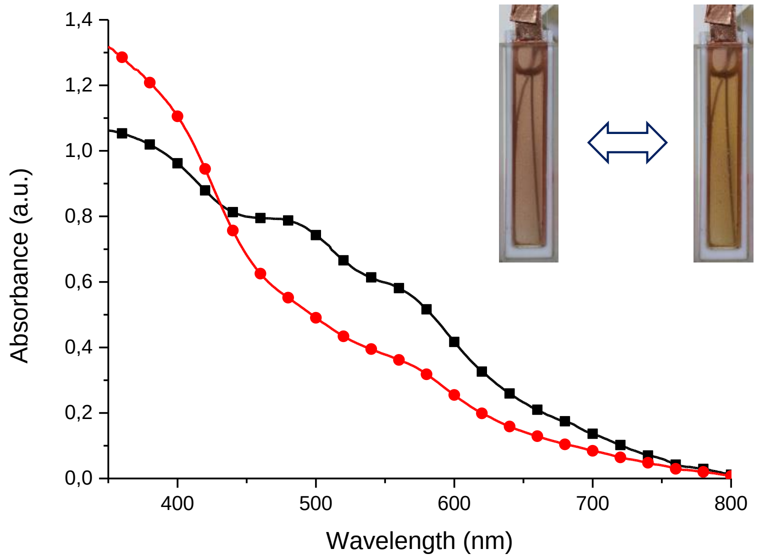

Figure S 32. UV-Vis spectra of polymer **P3** in its neutral (black) and electrochemically oxidized (red) states. Insert: photographs of the original (left), electrooxidized (right) **P3**.

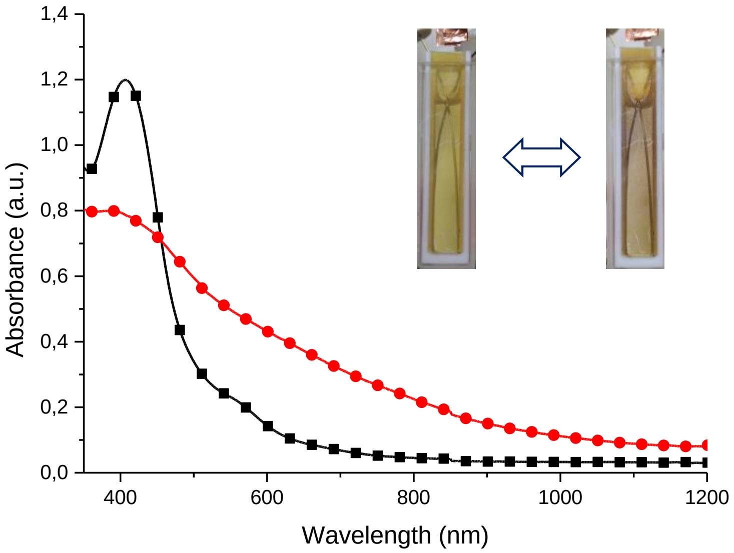

Figure S 33. UV-Vis spectra of polymer **P4** in its neutral (black) and electrochemically reduced (red) states. Insert: photographs of the original (left), electroreduced (right) **P4**.

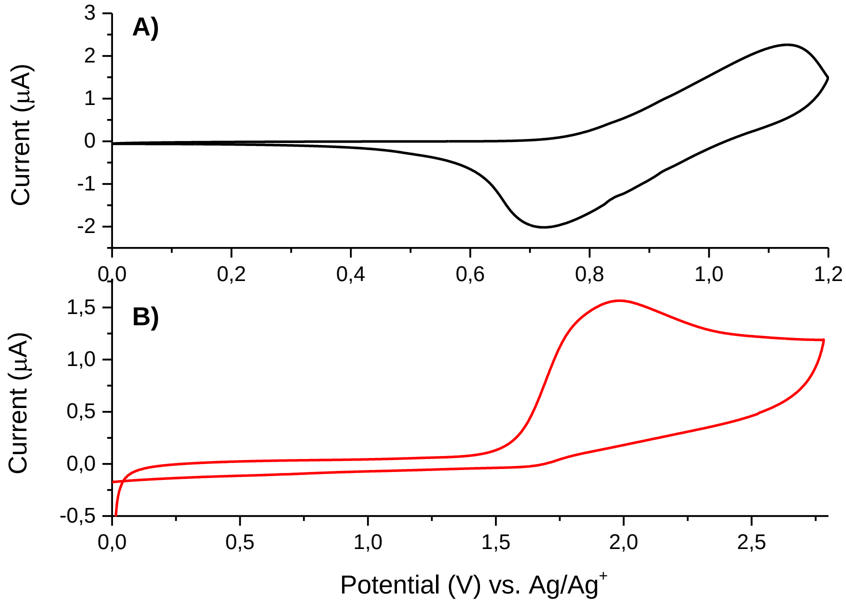

Figure S 34. Cyclic voltammogram thin films obtained by on-substrate polycondensation of **L** with A) dialdehyde **4** and B) dialdehyde **5** measured in anhydrous and deaerated 0.1 M solution of $TBAPF_6$ in acetonitrile.

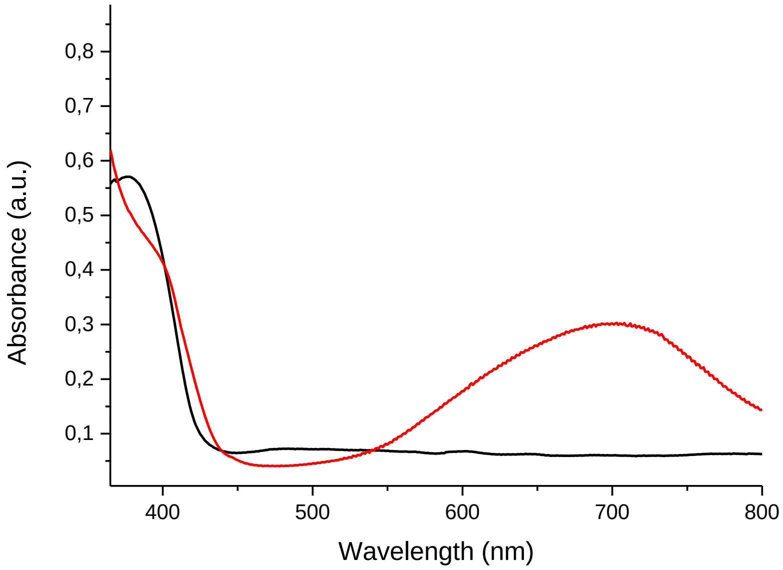

Figure S 35. UV-vis spectra of thin film obtained by polycondensation of **L** with dialdehyde **4** measured in anhydrous and deaerated 0.1M $TBAPF_6$ in acetonitrile as supporting electrolyte by applying 0 (black) and +1.2 V (red) potentials versus Ag/Ag^+ reference electrode held for 30 sec per potential.

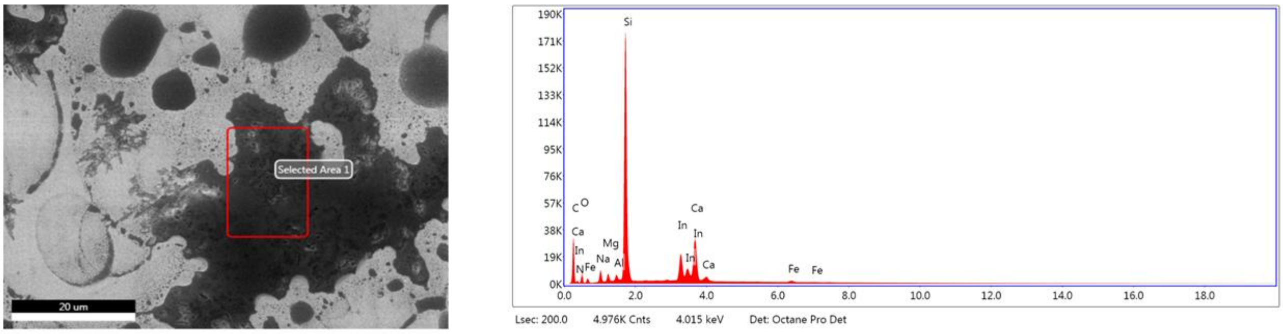

Figure S 36. Scanning Electron Microscopy (SEM) and EDX analysis of polymer **P1**.

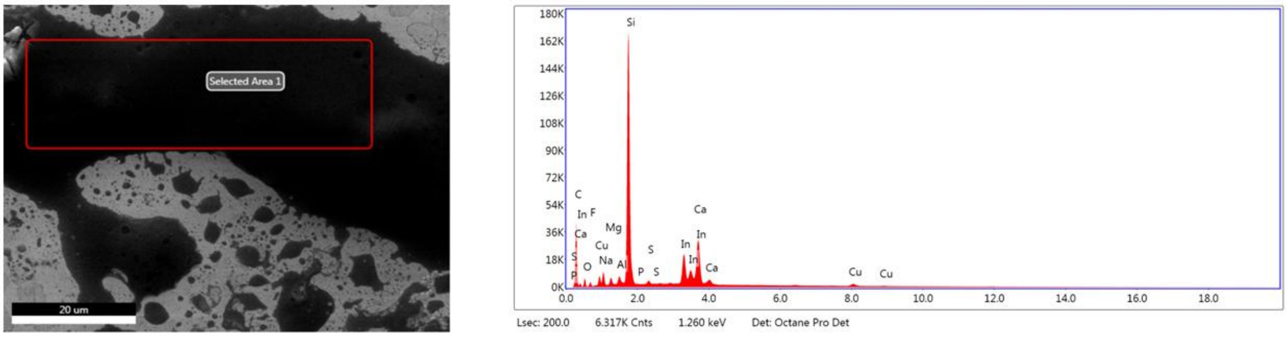

Figure S 37. Scanning Electron Microscopy (SEM) and EDX analysis of polymer **P2**.

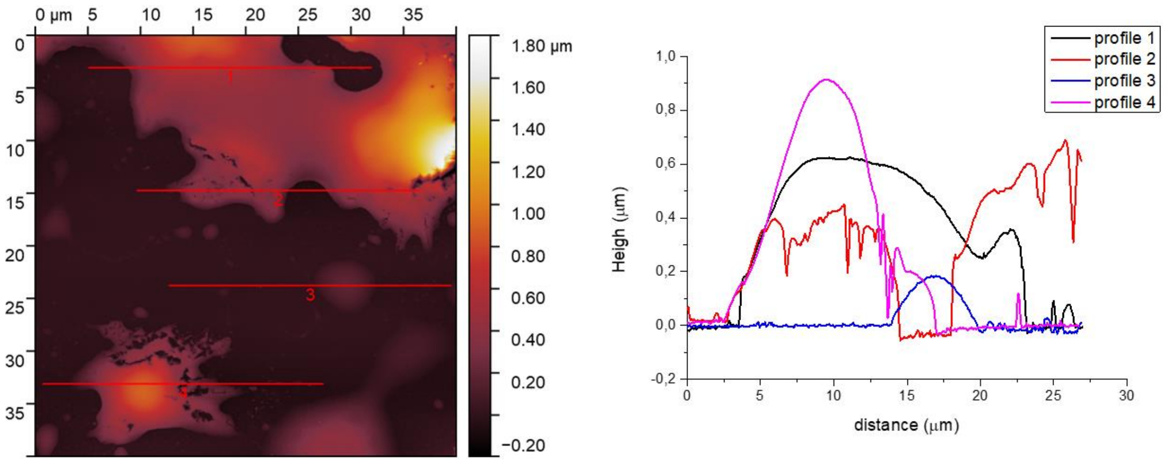

Figure S 38. AFM micrographs of polymer **P1** on ITO electrode. AFM cross-section profiles were measured at a marked places (left). AFM cross-section profiles of **P1** measured at marked places

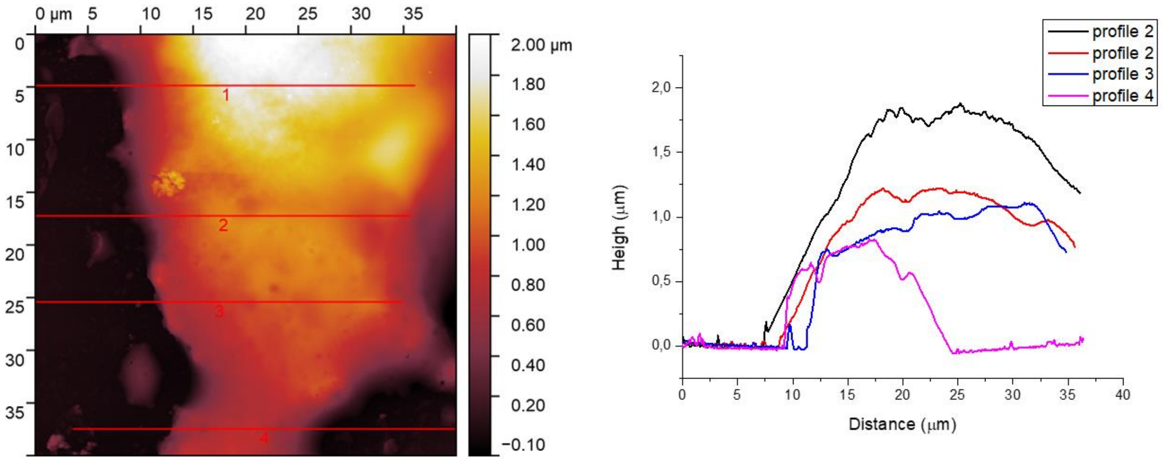

Figure S 39. AFM micrographs of polymer **P2** on ITO electrode. AFM cross-section profiles were measured at a marked places (left). AFM cross-section profiles of **P2** measured at marked places

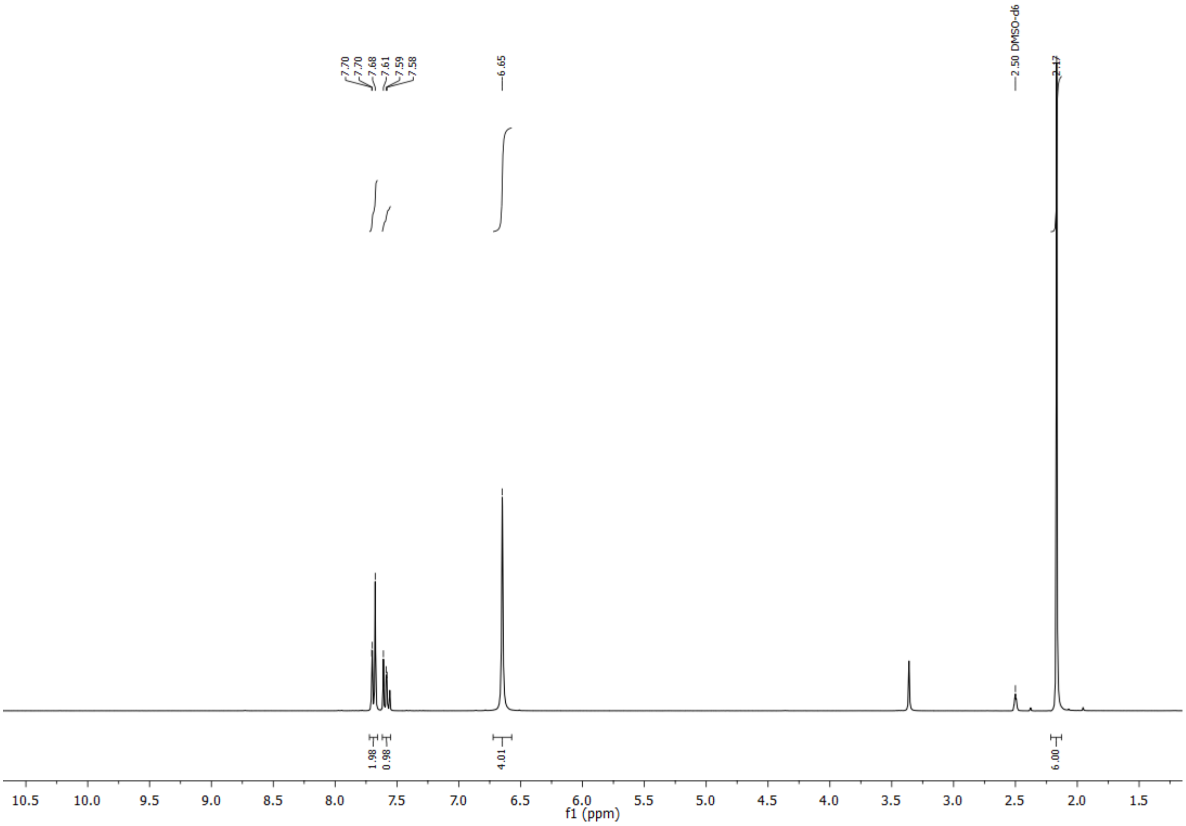

Figure S 40. ^{1}H NMR of ligand **L** in d_6-DMSO.

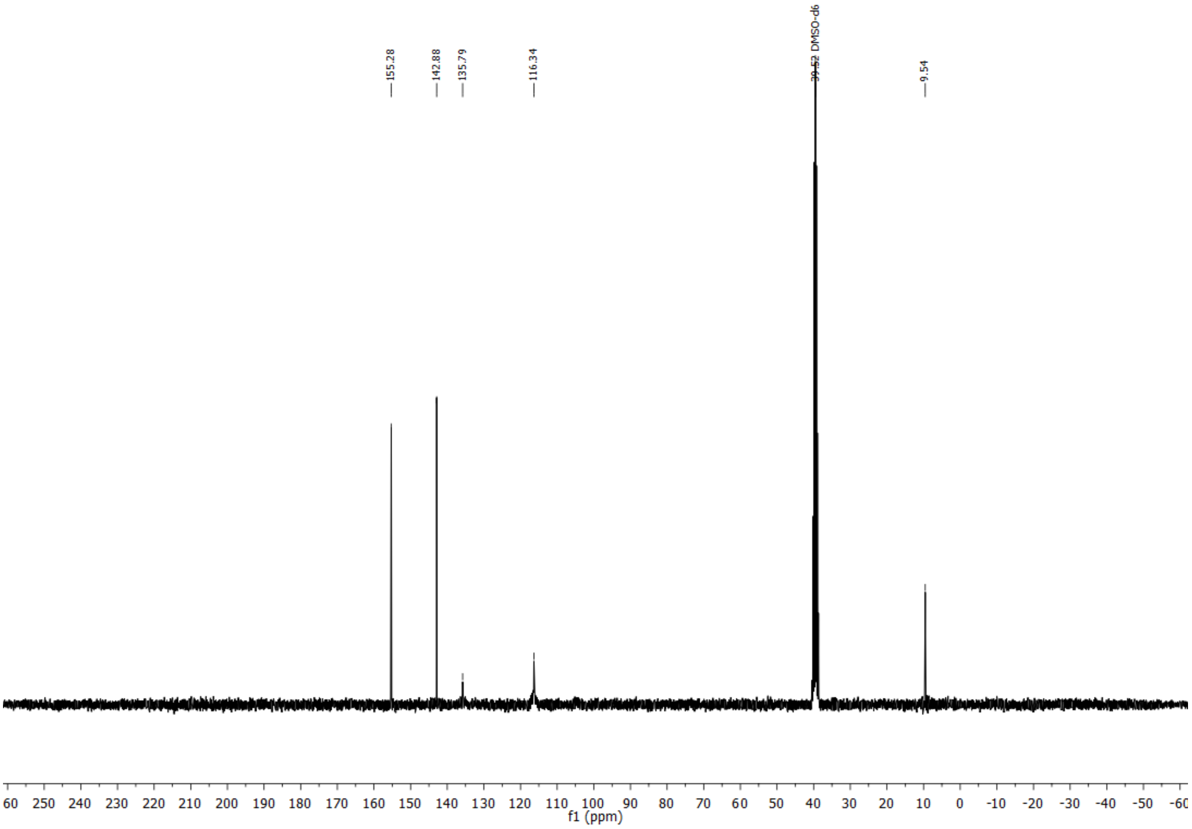

Figure S 41. ^{13}C NMR spectra of ligand **L**.

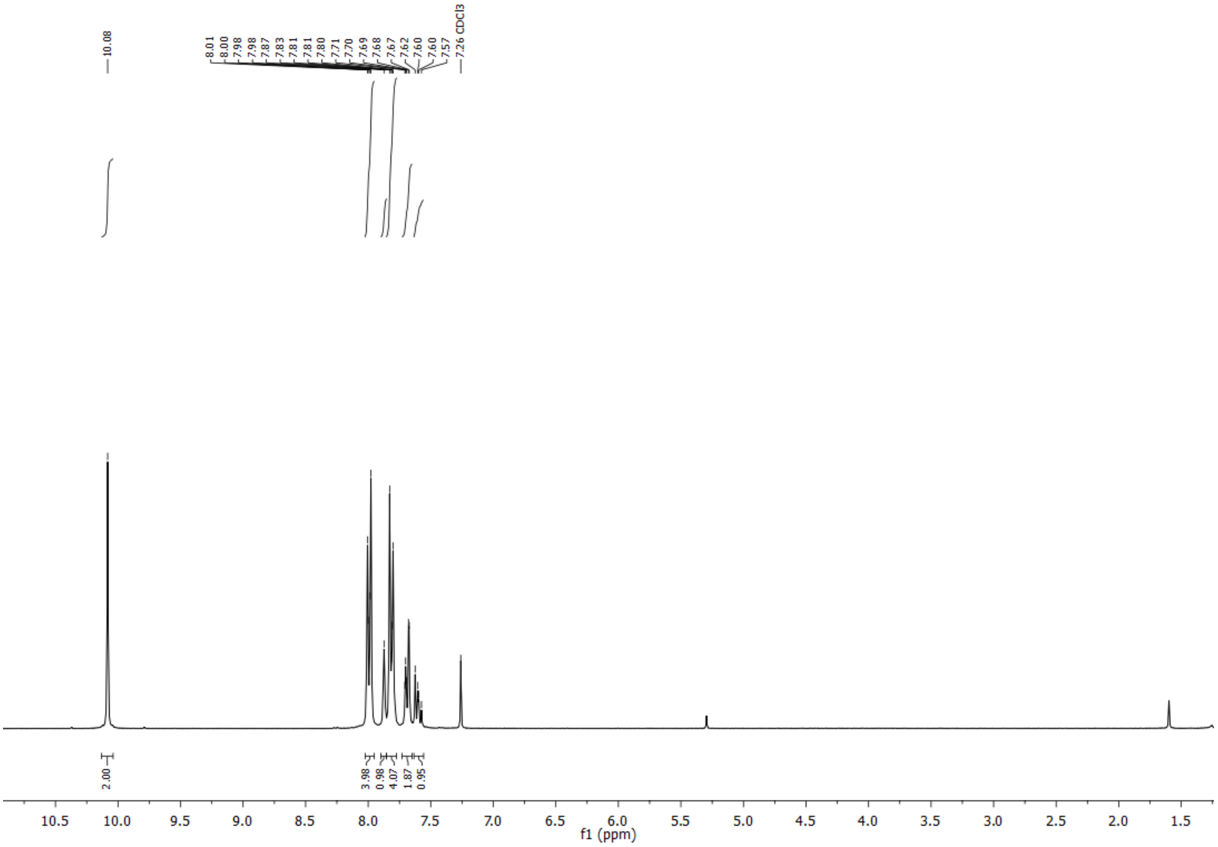

Figure S 42. ^{1}H NMR of dialdehyde **6** in $CDCl_3$.

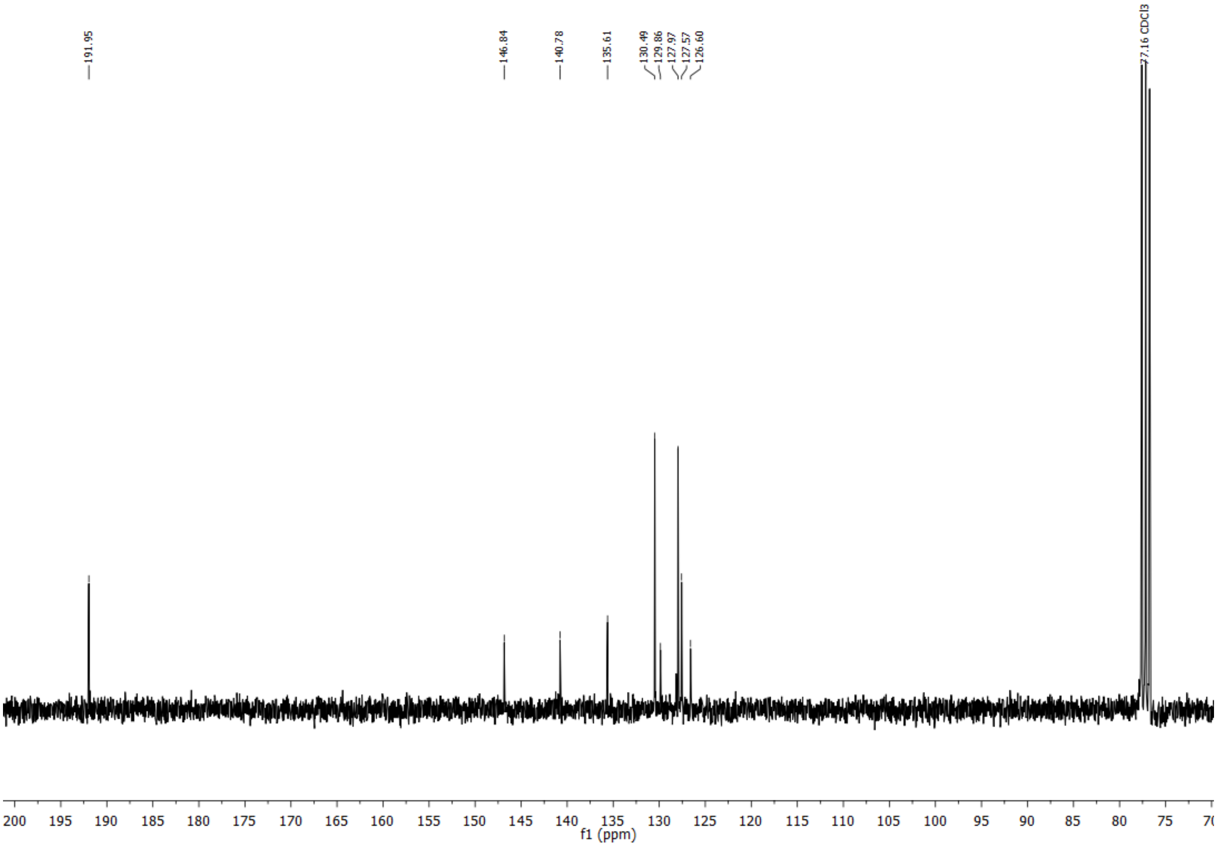

Figure S 43. ^{13}C NMR of dialdehyde **6** in $CDCl_3$.

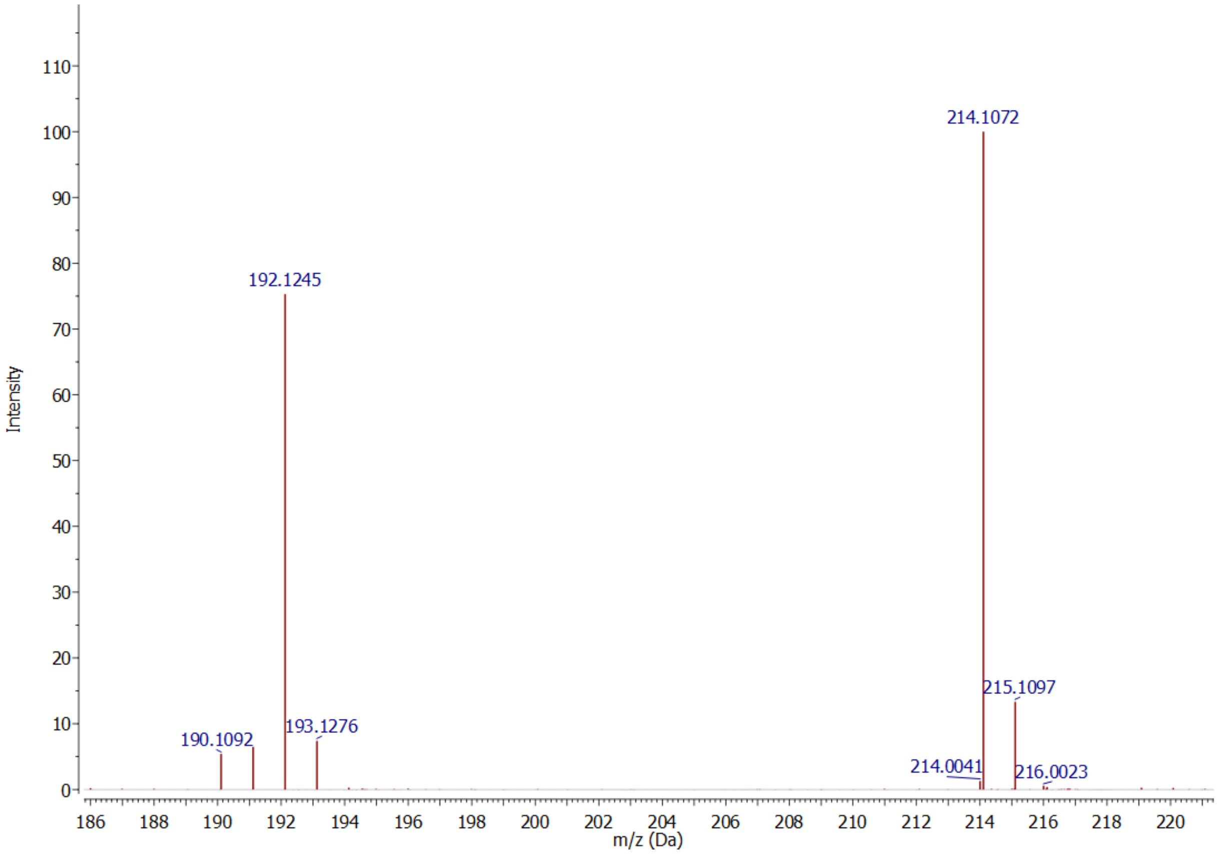

Figure S 44. HR-ESI-MS of ligand **L**.

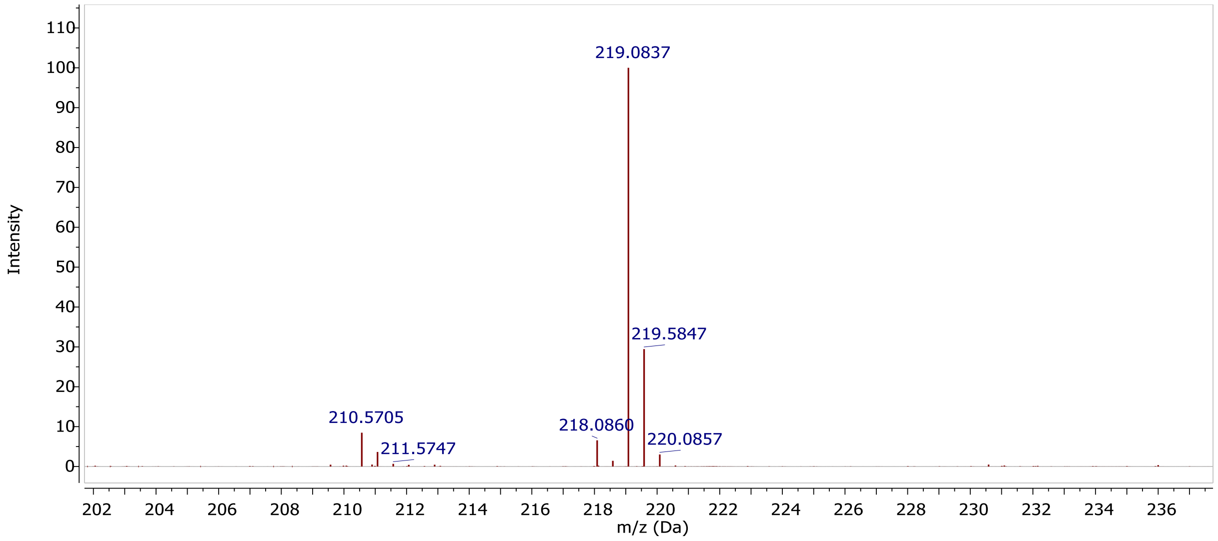

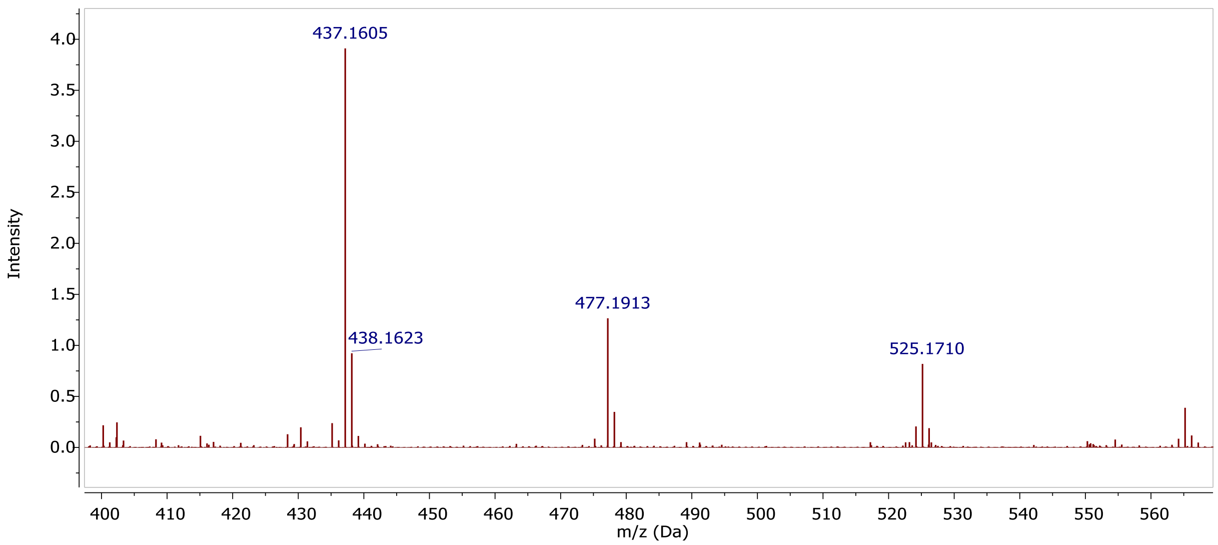

Figure S 45. HR-ESI-MS spectra of complex **1**.

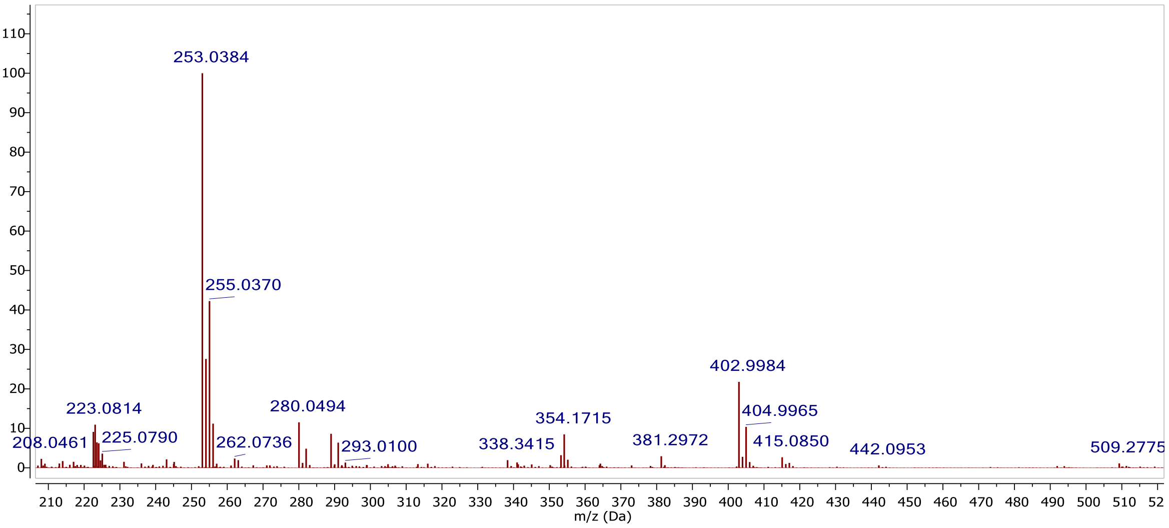

Figure S 46. HR-ESI-MS spectra of complex **2**.

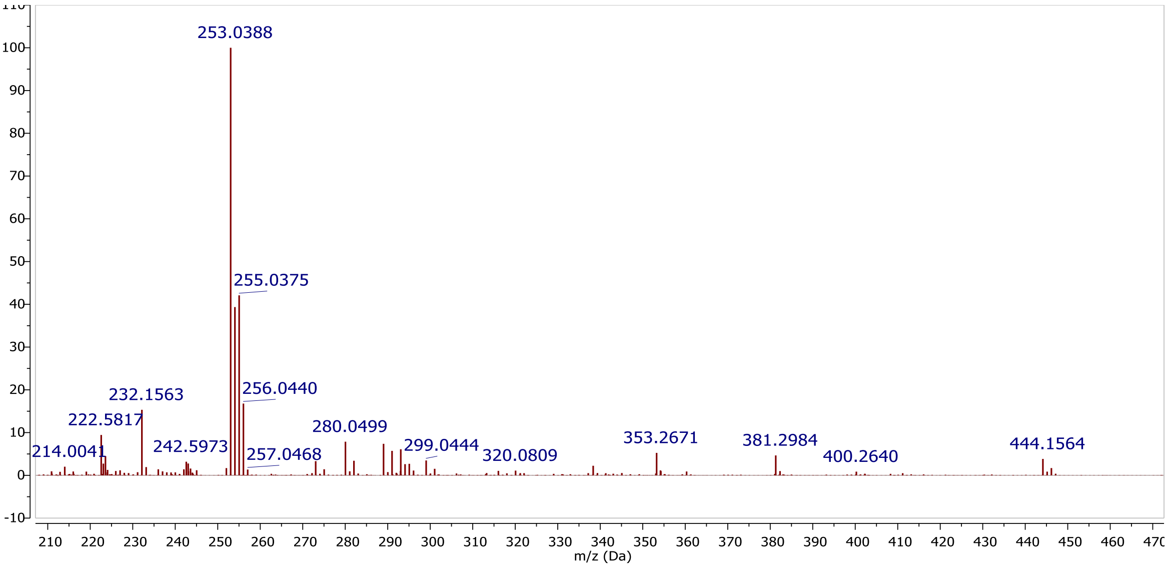

Figure S 47. HR-ESI-MS spectra of complex **3**.
